# A novel MOF-on-MOF composite versus its MOF shell: a comparative sorbent study for dispersive micro-solid phase extraction of pesticides in food samples

**DOI:** 10.1016/j.fochx.2025.103091

**Published:** 2025-09-27

**Authors:** Mahdi Ghorbani, Mojgan Ojaghzadeh Khalil Abad, Majid Keshavarzi

**Affiliations:** a Department of Chemistry, Faculty of Sciences, Ferdowsi University of Mashhad, Mashhad, Iran; b Department of Chemistry, Mashhad Branch, Islamic Azad University, Mashhad, Iran; c Department of Pharmacodynamics and Toxicology, School of Pharmacy, Mashhad University of Medical Sciences, Mashhad, Iran

**Keywords:** Organophosphorus pesticides, Dispersive micro-solid phase extraction, Comparative study, MOF on MOF composite, Vegetable samples, Commerial fruit juice samples

## Abstract

The pervasive application of organophosphorus pesticides (OPPs) in agriculture underscores the critical need for sensitive and reliable analytical techniques to safeguard food safety. Addressing this challenge, this work presents a rigorous comparative evaluation of two engineered sorbents for dispersive micro-solid phase extraction (D-μ-SPE) prior to gas chromatography–mass spectrometry (GC–MS): Bimetallic Fe/Co-MIL-88A and a novel Bimetallic Fe/Co-MIL-88A-on-MIL-88B composite. While both sorbents demonstrated high and statistically comparable extraction efficiencies, the composite was selected for its exceptional reusability and sustained performance over five consecutive cycles. Leveraging a design of experiments (DoE) approach, key extraction parameters were optimized for both sorbents, achieving remarkable sensitivity and precision. The method demonstrates an impressive linear dynamic range (LDR) of 0.07–900 ng mL^−1^, with detection limits as low as 0.02–0.09 ng mL^−1^, alongside high enrichment factors (up to 67) and excellent reproducibility (RSDs 3.16–4.35 %). Analysis of vegetable and fruit juice samples yielded robust recoveries (93.5–103.6 %) with precision (RSDs 4.37–6.33 %), affirming the method's reliability for routine monitoring. This work not only advances analytical capabilities for OPP detection but also contributes significantly to food safety surveillance, ensuring enhanced consumer protection.

## Highlights

• Bimetallic Fe/Co-MIL-88A-on-MIL-88B composite engineered for superior OPP adsorption capacity.
• GC–MS coupled with D-μ-SPE enables trace-level quantification of OPPs in complex matrices.
• Composite sorbent exhibits stable performance across five consecutive extraction cycles.
• Method achieves high enrichment factors for ultra-trace OPP determination.
• Robust precision ensures reliable monitoring in food safety protocols.

## 1 Introduction

Organophosphorus pesticides (OPPs) are potent neurotoxic insecticides widely used in agriculture to protect crops and enhance food production (Fu et al., 2022). However, their extensive application has led to widespread environmental contamination, resulting in OPP residues in soil, water, and ultimately, the food chain, raising significant concerns about human exposure (Liu et al., 2025; Zhang, Giesy, & Wang, 2022). OPPs exert their toxicity by irreversibly inhibiting acetylcholinesterase (AChE), a critical enzyme responsible for nerve impulse transmission (Aroniadou-Anderjaska, Figueiredo, de Araujo Furtado, Pidoplichko, & Braga, 2023; Ganie, Javaid, Hajam, & Reshi, 2022). This inhibition leads to acetylcholine accumulation and cholinergic overstimulation, causing a range of adverse health effects (Sridhar, 2021). Human exposure occurs through dietary intake, inhalation, and dermal contact, resulting in both acute (e.g., SLUDGE symptoms – Salivation, Lacrimation, Urination, Defecation, Gastrointestinal distress, and Emesis) and chronic health problems, including neurodevelopmental issues in children, neurological damage, respiratory ailments, and even potential links to certain cancers (Chen et al., 2024; Jaiswal et al., 2024; Sarailoo, Afshari, Asghariazar, Safarzadeh, & Dadkhah, 2022). Similar adverse effects are also observed in wildlife, highlighting the far-reaching ecological impact of these pesticides, disrupting ecosystem function and endangering vulnerable species (Ige, Aliu, Omole, & Alabi, 2025; Naz, Iqbal, Manan, Chatha, & Zia, 2023). Consequently, accurate and reliable determination of OPP residues in complex food matrices and environmental samples is essential for monitoring compliance with established Maximum Residue Limits (MRLs), enforcing regulations, and ultimately protecting public and environmental health. This necessitates the development of highly sensitive, selective, and efficient analytical methods, where sample preparation, particularly the extraction and preconcentration of OPPs, plays a crucial role.

The accurate determination of OPPs in complex food matrices presents a significant analytical challenge (Pan et al., 2022; Radowan, 2024). Raw food samples contain a diverse range of interfering compounds that can compromise the accuracy and sensitivity of analytical techniques such as gas chromatography–mass spectrometry (GC–MS) (Bhattu, Kathuria, Billing, & Verma, 2022). Consequently, efficient and selective sample preparation is crucial for achieving reliable quantification of OPP residues (Darvishnejad, Raoof, Ghani, & Ojani, 2023; Ghorbani et al., 2021a; Pan et al., 2022). Microextraction techniques, particularly dispersive micro solid-phase extraction (D-μ-SPE), have emerged as attractive alternatives to conventional methods, offering numerous advantages (Dugheri et al., 2021). These include reduced solvent consumption, a simplified and rapid procedure, enhanced sensitivity, minimized analyte loss, and high matrix compatibility (El-Deen, 2024). Importantly, D-μ-SPE achieves these benefits without relying on sophisticated and expensive instrumentation, making it an accessible and cost-effective approach that aligns with the principles of green chemistry and reduces environmental impact (Bedair et al., 2024). The performance of D-μ-SPE is critically dependent on the choice of sorbent material (D'Orazio, 2024). The sorbent must selectively extract OPPs from the complex food matrix while minimizing the co-extraction of interfering compounds such as lipids, pigments, and other pesticides (Veloo & Ibrahim, 2021). Therefore, careful selection of the appropriate sorbent, considering factors like surface area, pore size, chemical functionality, and selectivity towards specific OPPs, is paramount for achieving accurate and reliable quantification of OPP residues in food samples (Castañeda et al., 2024; Hashemi & Kaykhaii, 2024).

While a variety of analytical methods for OPP determination are indeed established, many rely on conventional sorbents—such as CuO nano plate-polyaniline composite, Magnetic graphene@Fe3O4@SiO2@TiO2 nanocomposites, cyanopropyl functionalized silica nanoparticles, or their combinations—in solid-phase extraction (SPE) or dispersive-SPE (d-SPE) workflows (Alizadeh, Mashalavi, Yeganeh Faal, & Seidi, 2021; Dozein, Masrournia, Es'haghi, & Bozorgmehr, 2021; Korrani, Khalili, Kamyab, Ibrahim, & Hashim, 2023). Although effective, these sorbents often suffer from limitations, including moderate selectivity leading to co-extraction of matrix interferents, limited reusability, and sometimes high solvent consumption during the elution or sorbent regeneration steps (Sanyal & Mathur, 2022). Furthermore, the development of new sorbent materials with enhanced properties—such as superior chemical stability, higher surface areas, and tailored porosity for improved selectivity—remains a vibrant and critical area of research (Patra, Dey, Solanki, Sengupta, & Mittal, 2025). This pursuit aims not only to push the boundaries of sensitivity and reduce environmental impact through greener methodologies but also to create more robust and cost-effective solutions for routine analysis. The ideal sorbent would offer high extraction efficiency, exceptional reusability to minimize cost and waste, and high matrix tolerance to ensure accurate quantification in complex samples like pigmented vegetables and fruit juices, which remain a significant analytical challenge (Jagirani & Soylak, 2024). It is within this context—addressing the limitations of current sorbent materials—that our work on developing a novel MOF-on-MOF composite finds its strong justification.

Metal-organic frameworks (MOFs) are a fascinating class of crystalline materials constructed from metal ions or clusters connected by organic linkers, forming highly porous structures with tunable pore sizes, shapes, and functionalities (Cai, Yan, Zhang, Zhou, & Jiang, 2021). Their exceptional surface area, high porosity, and chemical versatility make them outstanding candidates as sorbents in various applications, including D-μ-SPE (Ghorbani et al., 2024; Mane et al., 2024). MOFs offer several advantages over traditional sorbents in D-μ-SPE, including: enhanced selectivity through tailored pore sizes and chemical functionalities; higher extraction efficiency due to their exceptional surface area; and the potential for reusability and easy regeneration (Bedair et al., 2024; Ghorbani et al., 2024). The development of MOF-on-MOF composites represents a significant advancement in sorbent materials, offering synergistic properties that are particularly valuable for the extraction of OPPs (Gumus & Soylak, 2025). These hierarchical structures, where one MOF (the core) acts as a support for another (the shell), combine the advantages of both parent MOFs while mitigating their individual limitations (Haldar & Wöll, 2021). The core provides high surface area and structural stability, while the shell introduces specific functionalities tailored for enhanced OPPs recognition and capture (Zhang et al., 2021). For instance, a core MOF with high mechanical strength can be coated with a shell MOF decorated with functional groups that exhibit strong binding affinity towards OPPs. This strategic combination leads to novel sorbents with unique properties crucial for efficient OPPs extraction, including enhanced adsorption capacity and selectivity due to synergistic interactions; improved mass transfer kinetics facilitated by the hierarchical pore structure; and enhanced chemical and mechanical stability protecting the core MOF from degradation in complex sample matrices (Abad, Masrournia, & Javid, 2023; Alhmaunde, Masrournia, & Javid, 2022; Qin et al., 2023). Therefore, the rational design and synthesis of MOF-on-MOF composites are critical for addressing the challenges associated with OPPs extraction from environmental and food samples. The synthesis of the MIL-88B core in this work is adapted from established methods (Abad, Masrournia, & Javid, 2024). Building upon this, we engineered a novel hierarchical composite by growing a shell of MIL-88B onto the Fe/Co-MIL-88A core. While the individual components have been studied, the application of this specific Fe/Co-MIL-88A-on-MIL-88B composite as a sorbent material is presented here for the first time. Although this family of sorbents has demonstrated efficacy in extracting other classes of analytes, its unique properties—including the synergistic effect between the bimetallic core and the functionalized shell—are uniquely exploited in this study for the highly efficient and selective extraction of OPPs from challenging food matrices.

This study presents a streamlined and effective gas chromatography–mass spectrometry (GC–MS) method coupled with D-μ-SPE for the quantification of OPPs in a variety of vegetable and commercially available fruit juice matrices. This work presents a comprehensive comparative study of two novel sorbents: Bimetallic Fe/Co-MIL-88A and Bimetallic Fe/Co-MIL-88A on MIL-88B (MOF on MOF), which were synthesized via a bimetallic MOF approach for OPP extraction. The extraction efficiencies of these synthetic sorbents were statistically compared. A design of experiments (DoE) approach was implemented to optimize key parameters influencing OPP extraction for each sorbent individually. Crucially, both sorbents were carried through the entire methodological workflow to enable a rigorous evaluation of their operational performance, including stability and reusability in complex matrices, not just their initial extraction efficiency. Under optimized conditions, the performance of the developed method was evaluated by analyzing a range of vegetable and commercial fruit juice samples using both sorbents. A comprehensive comparison of the two sorbents' performance was conducted based on the analytical results. Finally, the merits of the proposed method were benchmarked against those of existing methodologies.

## 2 Experimental

### 2.1 Chemicals and reagents

Four OPPs, specifically chlorpyrifos, phosalone, fenitrothion, and profenofos, were obtained from Samiran Co. (Tehran, Iran). Iron(III) nitrate nonahydrate (≥98 %), iron(II) sulfate heptahydrate (≥99 %), cobalt(II) nitrate hexahydrate (98 %), terephthalic acid (98 %), and fumaric acid (≥99 %) were purchased from Sigma-Aldrich (USA). All solvents used in this study, including methanol (99 %), *N*,*N*-dimethylformamide (DMF) (≥99.8 %), ethylene glycol (≥99.5 %), ethanol (≥99.8 %), 1-propanol (≥99.8 %), acetone (≥99.8 %), ethyl acetate (≥99.8 %), and acetonitrile(≥99.8 %), were GC grade and acquired from Merck (Germany) or Sigma-Aldrich (USA).

### 2.2 Instrumental

The determination of OPPs – specifically, Fenitrothion, Chlorpyrifos, Profenofos, and Phosalone – was performed using an Agilent Gas chromatography (model 7890 A, USA) equipped with an MS detector. An HP-5 ms capillary column (Agilent 19091S-433, 30 m × 0.25 mm i.d., 0.25 μm film thickness) was employed for chromatographic separation. Helium was used as the carrier gas at a flow rate of 1 mL min^−1^. A 1 μL sample volume was injected into the GC–MS system in splitless mode. The oven temperature program was as follows: initial temperature of 95 °C held for 1.5 min, ramped to 192 °C at a rate of 20 °C min^−1^, then to 230 °C at 5 °C min^−1^, and finally to 290 °C at 25 °C min^−1^, and held for 20 min. The ion source temperature was maintained at 250 °C, and the interface temperature was set to 310 °C. Electron impact ionization (EI) at 70 eV was used for ionization. The mass spectrometer was operated in full scan mode, acquiring data over a mass-to-charge (*m*/*z*) range of 40–460. The morphology and structural characteristics of the MIL-88B and bimetallic Fe/Co-MIL-88A on MIL-88B composite were investigated using a suite of analytical techniques. X-ray diffraction (XRD) patterns were acquired using a Bruker D8 ADVANCE diffractometer (Germany). Microstructural analysis was performed via field-emission scanning electron microscopy (FE-SEM) using a Mira 3 Tescan instrument (Czech Republic). The specific surface area and pore size distribution were determined via nitrogen adsorption-desorption isotherms using a BELSORP Mini II analyzer (Japan) based on the Brunauer-Emmett-Teller (BET) theory.

### 2.3 Sorbent preparation

#### 2.3.1 MIL-88B as the core material

In a typical procedure, iron(II) sulfate heptahydrate (5.56 g, 0.02 mmol) and terephthalic acid (0.831 g, 0.005 mmol) were introduced into a 50 mL round-bottom flask containing a solution of DMF (20.0 mL) and ethylene glycol (5.0 mL). The resulting mixture was then subjected to vigorous stirring at 600 rpm for 60 min to ensure homogeneity. Subsequently, the homogeneous solution was transferred into a Teflon-lined stainless-steel autoclave and heated to 180 °C for 12 h under autogenous pressure. After the reaction, the autoclave was allowed to cool naturally to room temperature. The solid product was then separated by centrifugation at 5000 rpm for 10 min. The resulting precipitate was washed twice with ethanol to remove any residual reactants and byproducts. Finally, the obtained solid was dried in a conventional oven at 60 °C for 12 h (Abad et al., 2024).

#### 2.3.2 Bimetallic Fe/Co MIL-88A on MIL-88B as the Composite sorbent

In a typical synthesis, iron(III) nitrate nonahydrate (0.808 g, 2 mmol), cobalt(II) nitrate hexahydrate (0.582 g, 2 mmol), fumaric acid (0.58 g, 5 mmol), and MIL-88B (0.134 g) were introduced into a 100 mL round-bottom flask containing 30.0 mL of DMF. The resulting suspension was then subjected to a solvothermal reaction at 160 °C for 5 h under continuous stirring, using an oil bath to maintain a precise temperature. Following the reaction, the solid product was isolated by centrifugation at 4000 rpm for 10 min. The resulting precipitate was rigorously washed twice with DMF to remove unreacted precursors and byproducts, followed by further washing with methanol to facilitate solvent exchange and improve the subsequent drying process. The final product was dried at 80 °C in a conventional oven for 30 min to ensure complete solvent removal and achieve a stable, solvent-free material (Rabeie & Mahmoodi, 2024).

#### 2.3.3 Bimetallic Fe/Co MIL-88A as the Shell sorbent

To investigate and compare the extraction performance of the prepared sorbent for OPPs, bimetallic Fe/Co MIL-88A was synthesized according to the method described in Section 2.3.2, excluding the addition of MIL-88B.

### 2.4 Extraction procedure

An appropriate amount of prepared vegetable sample or filtered fruit juice (section 3.8) was transferred to a centrifuge tube. The pH of the sample was adjusted to its pre-determined optimal value for the specific sorbent used. An optimal amount of sodium chloride was added, followed by the addition of an optimal amount of either the Shell sorbent or the Composite sorbent. The resulting suspension was stirred at 500 rpm for its optimal extraction time to facilitate the adsorption of OPPs onto the sorbent material. After adsorption, the suspension was centrifuged at 5000 rpm for 7 min. The supernatant was discarded. To elute the adsorbed OPPs, 140 μL of acetonitrile was added to the sorbent, and the mixture was stirred at 500 rpm for 9 min. Following desorption, the mixture was centrifuged at 5000 rpm for 7 min. The acetonitrile extract was transferred to a clean vial and evaporated to near dryness under a gentle stream of nitrogen gas. The residue was reconstituted in 100 μL of ethyl acetate, and a 1 μL aliquot was injected into the GC–MS system for analysis.

## 3 Results and discussion

### 3.1 Sorbent characterization

**Fig. 1:**
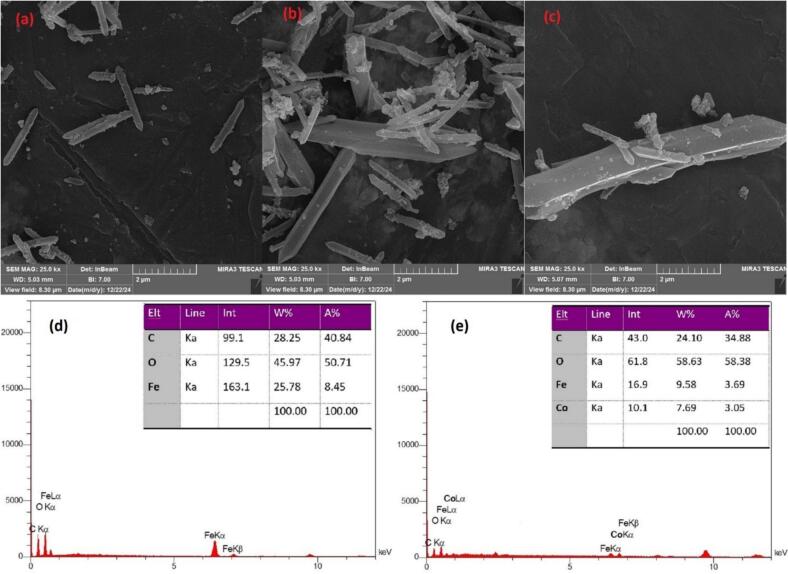
FESEM images of MIL-88B (a) and Composite sorbent (b and c) and EDX patterns of MIL-88B (d) and Composite sorbent (e).

Fig. 1 presents Field Emission Scanning Electron Microscopy (FE-SEM) images of the synthesized MIL-88B and the Composite sorbent, providing insights into their morphology and structure. The FE-SEM image of MIL-88B (Fig. 1a) reveals the presence of rod-shaped crystalline structures, which are characteristic of the MIL-88B metal-organic framework. These rods exhibit a relatively uniform size distribution and a smooth surface morphology, with lengths and widths typically in the range of 2.1 μm and 0.25 μm, respectively. The crystals appear to be well-defined and discrete, with minimal aggregation observed. In contrast, the FE-SEM images of the Composite sorbent (Fig. 1b and c) display a more complex morphology. At lower magnifications (Fig. 1b), the presence of larger, plate-like structures resembling aggregated material is evident. These plates are believed to be the MIL-88B, upon which a secondary phase consisting of smaller, rod-shaped particles (similar in morphology to the Composite sorbent crystals) is dispersed. Closer examination at higher magnification (Fig. 1c) confirms the presence of the Composite sorbent crystals on the surface of the MIL-88B matrix. The the Composite sorbent crystals appear to be well-distributed and adhered to the MIL-88B support, suggesting a successful deposition of the active phase onto the support material. The difference in structure between Fig. 1a and b may indicate that the structure of the sorbent surface have been changed, which might promote the adsorption of OPPs.

Energy-Dispersive X-ray Spectroscopy (EDX) analysis was performed to determine the elemental composition of the synthesized MIL-88B support and the Composite sorbent. The resulting EDX spectra and quantitative elemental analysis are presented in Figs. 1d and e. The EDX spectrum of MIL-88B (Fig. 1d) exhibits characteristic peaks corresponding to carbon (C), oxygen (O), and iron (Fe). Quantitative analysis reveals that the material is composed of 28.25 wt% carbon, 45.97 wt% oxygen, and 25.78 wt% iron. The presence of these elements is consistent with the expected elemental composition of MIL-88B, which consists of iron(III) oxide chains coordinated by fumarate ligands. The EDX spectrum of the Composite sorbent (Fig. 1e) shows peaks corresponding to carbon (C), oxygen (O), iron (Fe), and cobalt (Co). Quantitative analysis indicates the presence of 24.10 wt% carbon, 58.63 wt% oxygen, 9.58 wt% iron, and 7.69 wt% cobalt. The presence of both iron and cobalt confirms the successful incorporation of the bimetallic Fe/Co component into the Composite sorbent. Also, a decrease in iron concentration and a high concentration of oxygen indicate the use of MIL-88B. These results are consistent with the intended composition of the materials, confirming that the bimetallic composite material contains iron and cobalt, indicating successful doping into the Composite sorbent.

**Fig. 2:**
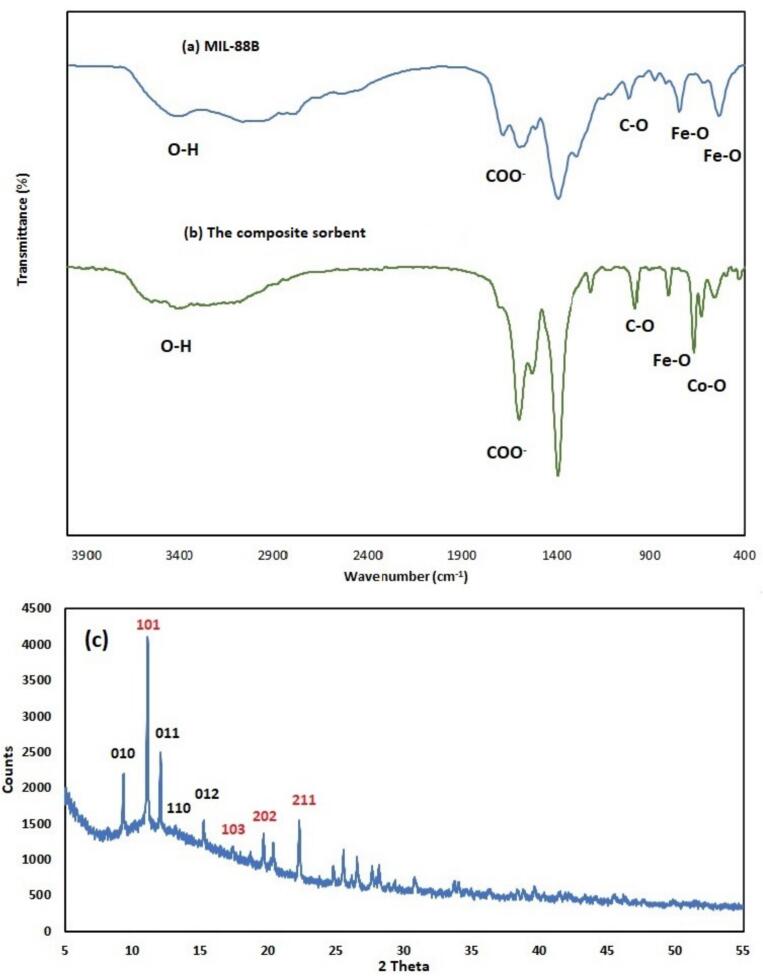
The FTIR spectra of (a) MIL-88B and (b) composite sorbent; (c) XRD pattern of composite sorbent. Miller indices are shown in red for the MIL-88B structure and in black for the bimetallic Fe/Co-MIL-88A phase. (For interpretation of the references to colour in this figure legend, the reader is referred to the web version of this article.)

The FTIR spectra of MIL-88B and Composite sorbent are presented in Fig. 2a and b, respectively. A broad absorption band centered at 3404 cm^−1^ in MIL-88B (Fig. 2a) corresponds to O—H stretching vibrations from hydroxyl groups or coordinated water molecules within the metal-organic framework (MOF). In the Composite sorbent (Fig. 2b), this peak shifts slightly to 3423 cm^−1^, indicating altered hydrogen bonding interactions or partial dehydration induced by cobalt incorporation. In MIL-88B, the asymmetric stretching vibrations of carboxylate groups are observed at 1684 cm^−1^ and 1597 cm^−1^, consistent with bidentate coordination of organic ligand (terephthalate) to Fe^3+^ centers. Upon cobalt integration (Fig. 2b), these peaks shift to 1604 cm^−1^ and 1534 cm^−1^, reflecting modified ligand coordination environments due to the formation of mixed Fe/Co secondary building units (SBUs). The symmetric carboxylate stretching vibration at 1388 cm^−1^ in MIL-88B, indicative of balanced metal-ligand charge distribution, remains nearly unchanged at 1397 cm^−1^ in the bimetallic system, suggesting retained carboxylate-metal interactions. The peak at 1016 cm^−1^ in MIL-88B is attributed to C—O stretching vibrations of the organic linker or framework modes. Below 1000 cm^−1^, the spectrum of MIL-88B exhibits characteristic Fe—O vibrations at 748 cm^−1^ and 532 cm^−1^, confirming Fe^3+^-based SBUs. In contrast, the Composite sorbent (Fig. 2b) shows distinct Co—O vibrations at 801 cm^−1^ and 430 cm^−1^, alongside Fe—O vibrations at 630 cm^−1^ and 559 cm^−1^, which collectively validate the coexistence of Fe and Co in the framework. These spectral changes—specifically, the shifts in carboxylate stretching modes and the emergence of Co—O vibrations—demonstrate structural reorganization within the MOF due to cobalt integration. The results confirm the successful synthesis of the Composite sorbent, highlighting synergistic interactions between Fe and Co centers that may enhance the material's adsorption properties.

The XRD pattern of the Composite sorbent is presented in Fig. 2C. The peaks at 2θ of 11.7, 17.3, 19.6, and 22.2 degrees with Miller indices (1 0 1), (1 0 3), (2 0 2), and (2 1 1), respectively, with a unit of counts, are attributed to the crystalline MIL-88B structure (Zorainy, Kaliaguine, Gobara, Elbasuney, & Boffito, 2022). Conversely, the peaks at 2θ of 9.3, 12.02, 13.2, and 15.2 degrees with Miller indices (0 1 0), (0 1 1), (1 1 0), and (0 1 2), respectively, indicate the presence of bimetallic Fe/Co-MIL-88A in the composite material (Ke et al., 2016). A broad hump between 5 and 10 degrees 2θ suggests the presence of some amorphous material or regions of lower crystallinity within the sample.

The textural properties of MIL-88B and the Composite sorbent were investigated using nitrogen adsorption-desorption isotherms. The MIL-88B exhibited a BET surface area of 4.14 m^2^g^−1^ and a Langmuir surface area of 118.34 4.14 m^2^g^−1^. The total pore volume for MIL-88B was determined to be 0.025 cm^3^ g^−1^, with a mean pore diameter of 24.11 nm. In contrast, the Composite sorbent showed significantly higher values, with a BET surface area of 26.19 m^2^g^−1^ and a Langmuir surface area of 345.85 m^2^g^−1^. The total pore volume for this composite was 0.156 cm^3^ g^−1^, and the mean pore diameter was 23.89 nm. The BJH plot analysis determined a peak radius area for MIL-88B of 4.903 m^2^g^−1^ at pore volume 0.025 cm^3^ g^−1^, with a corresponding peak radius of 1.85 nm. On the other hand, the Composite sorbent determined a peak radius area of 18.11 m^2^g^−1^ at a pore volume of 0.152 cm^3^ g^−1^, with a corresponding peak radius of 1.21 nm. These results indicate that the incorporation of Composite sorbent support leads to a substantial increase in the surface area and pore volume of the resulting material, potentially enhancing its adsorption capacity for OPPs extraction. The higher surface area and pore volume of the Composite sorbent suggest increased accessibility to active sites for OPPs binding.

### 3.2 Sorbent selection

To evaluate the extraction efficiencies of three sorbent materials – MIL-88B as the Core material, bimetallic Fe/Co MIL-88A as the Shell sorbent, and bimetallic Fe/Co MIL-88A supported on MIL-88B as the Composite sorbent – for the extraction of OPPs, a series of controlled experiments was conducted. The extraction parameters, including OPP concentration (50 ng mL^−1^), sorbent mass (30 mg), pH (7.0 ± 0.5), and extraction time (10 min), were held constant to ensure a fair comparison across the sorbents. This approach minimizes the influence of extraneous variables, allowing for a more accurate assessment of the inherent extraction capabilities of each material. The desorption process was carried out using 150 μL of methanol as the eluent, with sonication for 10 min to facilitate efficient analyte recovery. Methanol was chosen due to its high elution strength for OPPs, and then it was dried, and the residue was dissolved in ethyl acetate(100 μL) prior to GC–MS. Each extraction and desorption experiment was performed in triplicate to ensure reproducibility and permit statistical analysis. The resulting data, presented in Table 1, were subjected to rigorous statistical analysis at a 95 % confidence level. A Shapiro-Wilk test was employed to assess the normality of the data distribution for each sorbent. The Shapiro-Wilk test is particularly suitable for smaller sample sizes and provides a robust assessment of normality. The results (Table S1) revealed that the data obtained for each sorbent material followed a normal distribution (*p*-value >0.05), validating the use of parametric statistical methods for subsequent analysis. Specifically, Tukey's Honestly Significant Difference (HSD) test was used to compare the mean extraction efficiencies of the three sorbents (Table S2). The Tukey's HSD test indicated a statistically significant difference in OPP extraction efficiency between MIL-88B as the Core material and both the Shell and Composite sorbents. However, no statistically significant difference was observed between the Shell and Composite sorbents. Based on these findings, the Shell and Composite sorbents exhibited superior extraction performance compared to the unmodified MIL-88B. Therefore, the Shell and Composite sorbents were selected as the sorbent for further studies.

**Table 1:** Effect of the sorbent type for the OPP extraction.

Run	MIL-88B	Shell sorbent	Composite sorbent
1	59.63	79.71	87.39
2	66.24	82.32	82.65
3	64.71	84.56	84.22
Mean ± SD	63.53 ± 3.46	82.20 ± 2.43	84.75 ± 2.41

### 3.3 Desorption solvent type

The selection of a desorption solvent in D-μ-SPE for OPPs is of paramount importance as it directly influences the extraction efficiency and selectivity towards target analytes. An optimal desorption solvent should facilitate the efficient elution of retained OPPs from the sorbent material while minimizing the co-extraction of interfering matrix components, thereby maximizing sensitivity and accuracy in subsequent analytical techniques. Rigorous selection, optimization, and justification of the chosen desorption solvent are therefore critical for ensuring the reliability and reproducibility of experimental findings. In this study, methanol, ethanol, 2-propanol, acetone, acetonitrile, and ethyl acetate were evaluated as potential desorption solvents (Table 2). To mitigate potential issues associated with direct solvent injection into GC–MS and to enhance method reproducibility, the desorption solvent was evaporated under a gentle stream of nitrogen gas following the washing step. The resulting residue was then reconstituted in 100 μL of ethyl acetate prior to GC–MS analysis. The results were analyzed by statistical method at a 95 % confidence level using a Shapiro-Wilk test to assess the normality of the data distribution and Tukey's Honestly Significant Difference (HSD) test to compare the mean extraction efficiencies of OPPs (Table S3 and S4). Early findings indicated that acetonitrile (MeCN) offered strong extraction capabilities with a significant difference from other desorption solvents (*p*-value <0.05) using both sorbents. While acetonitrile's effectiveness can be attributed to its favorable polarity, enabling solvation of OPPs containing aromatic rings with moderate polarity. Furthermore, acetonitrile's capacity to interact with the polar functionalities of fumaric acid, the ligand within MIL-88A, could potentially disrupt secondary interactions between the OPPs and the sorbent framework, promoting analyte release. The relatively high affinity of acetonitrile for active sites on both sorbent surfaces may facilitate competitive displacement of OPPs, leading to enhanced elution. However, Ethyl acetate exhibits similar properties to acetonitrile for the extraction of OPPs. In contrast, while methanol and ethanol possess high polarity, their strong hydrogen-bonding capabilities could lead to excessive interactions with the fumaric acid ligand, hindering their ability to disrupt OPP-sorbent interactions effectively. Moreover, these solvents exhibit a greater propensity for extracting polar matrix interferences, potentially complicating the subsequent analysis. 2-Propanol shares similar characteristics with methanol and ethanol, but its longer alkyl chain results in a slight reduction in polarity and solvating power for moderately polar aromatic OPPs. The enhanced hydrophobic character may also impede efficient elution. Acetone, although a widely used solvent, exhibits lower polarity compared to acetonitrile and the alcohols. Consequently, its ability to effectively solvate the polar portions of OPPs or to compete for binding sites on the sorbent may be compromised. Therefore, acetonitrile was selected as the optimum desorption solvent for further studies.

**Table 2:** Effect of the desorption solvent type for the OPP extraction.

Desorption solvent	Shell sorbent		Composite sorbent	
	ER%	Mean ± SD	ER%	Mean ± SD
Methanol	79.71	82.20 ± 2.43	87.39	84.75 ± 2.41
	82.32		82.65	
	84.56		84.22	

Ethanol	83.21	80.98 ± 2.28	85.62	83.00 ± 2.38
	78.65		82.43	
	81.07		80.96	

2-Propanol	77.43	79.28 ± 1.64	82.03	80.72 ± 2.04
	79.87		81.75	
	80.55		78.37	

Acetonitrile	88.74	88.60 ± 1.78	94.81	91.99 ± 2.53
	90.31		91.24	
	86.75		89.92	

Ethyl acetate	81.19	82.21 ± 2.84	80.53	81.22 ± 3.17
	85.42		84.67	
	80.03		78.45	

Acetone	75.46	73.31 ± 2.44	76.88	74.06 ± 1.64
	73.81		74.08	
	70.65		71.22	

### 3.4 Screening design

Given the multi-factorial nature of analyte extraction, a Definitive Screening Design (DSD) was employed to efficiently identify significant factors influencing the extraction of OPPs. The DSD, comprising 17 experimental runs conducted in a randomized order to mitigate systematic bias, enabled the assessment of multiple factors with a relatively small number of experiments. Each experimental condition was performed in triplicate to provide a robust estimate of the response variable and to allow for quantification of experimental error. The specific factors examined, the design matrix, and the corresponding experimental responses for both sorbents are summarized in Table 3. Statistical analysis of the responses was performed using a one-way Analysis of Variance (ANOVA) at a significance level of α = 0.05, corresponding to a 95 % confidence interval. Factors exhibiting a *p*-value lower than 0.05 were deemed statistically significant, indicating a notable influence on the extraction efficiency of OPPs (Table S5). The overall model used for screening the factors was considered statistically significant, as evidenced by a model *p*-value below 0.05, suggesting that the selected factors, as a whole, significantly affect the extraction process. The ANOVA results revealed that sorbent amount, extraction time, and desorption solvent volume exerted a statistically significant impact (*p* < 0.05) on OPP extraction using both sorbents. Consequently, these three factors were selected for further optimization to determine the optimal conditions for maximizing OPP extraction. Conversely, sample volume, pH, desorption time, and salt percentage (weight percentage of sodium chloride) were identified as non-significant factors within the tested range of values, indicating a minimal influence on OPP extraction. Therefore, these factors were held constant at appropriate values during the subsequent optimization step to reduce experimental complexity and focus on the significant variables. The selected constant values for the non-significant factors were determined and are visualized in Fig. S2, where the red point indicates the chosen value for each factor. Specifically, the constant values of sample volume, pH, desorption time, and salt percentage were 12.0 mL, 7.4, 8.6 min, and 3.9 %, using the shell sorbent, and 19.0 mL, 7.6, 7.7 min, and 4.8 % using the composite sorbent, respectively.

**Table 3:** Screening design for OPP extraction using the Shell or Composite sorbents.

Factor	Name	Units	Minimum	Maximum	Coded Low	Coded High	Mean	Std. Dev.
A	pH	–	5.00	8.00	−1 ↔ 5.00	+1 ↔ 8.00	6.50	1.40
B	Sorbent mass	mg	20.00	40.00	−1 ↔ 20.00	+1 ↔ 40.00	30.00	9.35
C	Sample volume	mL	10.00	20.00	−1 ↔ 10.00	+1 ↔ 20.00	15.00	4.68
D	Extraction time	min	5.00	10.00	−1 ↔ 5.00	+1 ↔ 10.00	7.50	2.34
E	Desorption time	min	5.00	10.00	−1 ↔ 5.00	+1 ↔ 10.00	7.50	2.34
F	Desorption solvent volume	μL	100.00	300.00	−1 ↔ 100.00	+1 ↔ 300.00	200.00	93.54
G	Salt percentage	*w*/*v*%	0.0000	5.00	−1 ↔ 0.00	+1 ↔ 5.00	2.50	2.34


Std	Run	A	B	C	D	E	F	G	ER% using the Shell sorbent	ER% using the Composite sorbent
9	1	1	1	−1	−1	0	1	1	61.54	55.68
7	2	1	−1	−1	0	1	1	-1	51.72	59.33
16	3	-1	-1	-1	1	-1	1	1	55.19	51.63
11	4	1	-1	1	-1	-1	0	1	47.02	54.41
3	5	1	0	1	1	-1	1	-1	68.23	65.35
1	6	0	1	1	1	1	1	1	93.95	97.44
12	7	-1	1	-1	1	1	0	-1	92.87	88.71
10	8	-1	-1	1	1	0	-1	-1	73.98	73.64
17	9	0	0	0	0	0	0	0	70.45	66.45
14	10	-1	-1	1	-1	1	1	0	28.54	29.97
4	11	-1	0	-1	-1	1	-1	1	62.03	58.91
2	12	0	-1	-1	-1	-1	-1	-1	56.44	48.6
13	13	1	1	-1	1	-1	-1	0	91.34	93.71
6	14	-1	1	0	-1	-1	1	-1	57.97	51.82
5	15	1	-1	0	1	1	-1	1	90.45	85.79
15	16	1	1	1	-1	1	-1	-1	86.69	81.05
8	17	-1	1	1	0	-1	-1	1	92.56	88.15

### 3.5 Optimization design

**Fig. 3:**
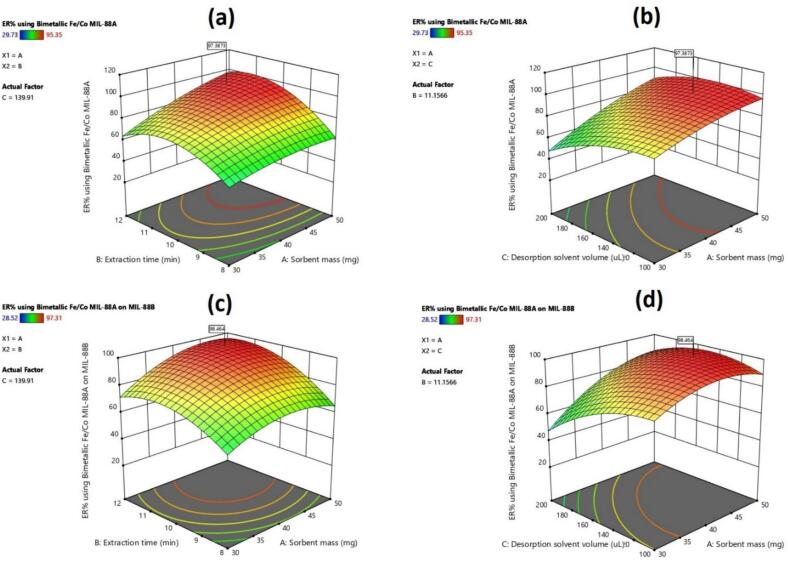
Significant binary interactions between factors for determining OPPs using the Shell sorbent (a and b) and the Composite sorbent (c and d).

A Central Composite Design (CCD) was implemented to optimize the three significant factors identified in the screening stage: sorbent mass, extraction time, and desorption solvent volume. The CCD comprised 20 experimental runs, randomized to minimize systematic bias. Each experimental condition was performed in triplicate to provide a robust estimate of the response variable and to enable the quantification of experimental error. Table 4 summarizes the design matrix, the factor levels, and the corresponding experimentally obtained responses. Statistical analysis of the responses was conducted using a ANOVA at a significance level of α = 0.05. The statistical parameters are presented in Table S6. The model was considered statistically significant, as indicated by a model *p*-value below 0.05, demonstrating that the selected factors, as a whole, significantly influence the extraction process. Furthermore, the non-significant lack-of-fit (*p* > 0.05) suggests that the model adequately captures the relationship between the factors and the response, without significant unexplained variance. The meaningful factor will affect the extraction and increase the extraction recovery, and it does not explain by error. Consistent with the screening stage, all three factors (sorbent mass, extraction time, and desorption solvent volume) exhibited a statistically significant impact (*p* < 0.05) on OPP extraction using both sorbents. Furthermore, the analysis revealed statistically significant binary interactions between sorbent mass and extraction time, and between sorbent mass and desorption solvent volume. Fig. 3 illustrates the effects of these significant binary interactions on the ER%. Fig. 3a and c indicate that ER% increased with simultaneous increases in both sorbent mass and extraction time using both sorbents. Also, an increase in sorbent mass and reduction in desorption solvent volume lead to an increase in the ER% (Fig. 3b and d). This observation aligns with the understanding that an increased sorbent mass provides a greater number of active sites for interaction with OPPs. Additionally, a minimum extraction time is necessary to achieve equilibrium adsorption of OPPs onto the sorbent surface. Also, ER% was reduced with an increase in desorption solvent volume, which aligns with the fact that increasing the volume of the liquid translates to a low concentration and dilutes the desorbed analytes in the desorption solvent.

**Table 4:** Central composite design for OPP extraction using the Shell or Composite sorbents.

Std	Run	A:Sorbent mass	B:Extraction time	C:Desorption solvent volume	ER% for the Shell sorbent	ER% for the Composite sorbent
7	1	-1	1	1	39.61	42.75
10	2	1	0	0	88.82	85.26
12	3	0	1	0	77.94	88.18
3	4	-1	1	-1	68.47	77.09
15	5	0	0	0	92.53	94.46
4	6	1	1	-1	95.35	89.51
19	7	0	0	0	94.07	92.51
2	8	1	-1	-1	59.31	60.47
17	9	0	0	0	89.46	90.39
9	10	-1	0	0	71.75	73.08
16	11	0	0	0	91.12	93.72
14	12	0	0	1	67.49	71.18
13	13	0	0	-1	85.43	88.55
18	14	0	0	0	89.95	97.31
8	15	1	1	1	88.33	88.41
20	16	0	0	0	90.83	92.14
6	17	1	-1	1	52.29	55.37
5	18	-1	-1	1	29.73	28.52
11	19	0	-1	0	57.98	66.61
1	20	-1	-1	-1	58.59	63.72

Conversely, the interaction between extraction time and desorption solvent volume exhibited a p-value exceeding 0.05, indicating a non-significant effect on analyte extraction.

The CCD analysis generated a second-order polynomial model to estimate the relationship between the independent variables and the response (ER%). The resulting quadratic equations are presented below:

(2) $$\mathrm{ER}\%\text{for the Shell sorbent}=88.5036+11.595^{*}\;A+11.18^{*}\;B–8.97^{*}\;C+6.54^{*}\;\mathrm{AB}+5.46^{*}\;\mathrm{AC}+2.5121\text{5e}-15^{*}\;\mathrm{BC}-3.98409^{*}\;A^{2}–16.3091^{*}\;B^{2}–7.80909^{*}\;C^{2}$$

(3) $$\mathrm{ER}\%\text{for the composite sorbent}=91.4372+9.386^{*}\;A+11.125^{*}\;B–9.311^{*}\;C+4.31^{*}\;\mathrm{AB}+7.9175^{*}\;\mathrm{AC}+0.6075^{*}\;\mathrm{BC}-9.29045^{*}\;A^{2}–11.0655^{*}\;B^{2}–8.59545^{*}\;C^{2}$$

(where A = sorbent mass, B = extraction time, and C = desorption solvent volume)

The statistical parameters for the fitted equations are presented in Table S7. The equations demonstrated a good fit to the experimental data, as evidenced by high R^2^ values (0.9715 and 0.9798) and adjusted R^2^ values (0.9459 and 0.9615) for the Shell and Composite sorbents, respectively. Furthermore, high and proper predicted R^2^ values (0.8737 and 0.9089) confirmed that the model exhibits a suitable ability to predict the response within the experimental design space for both sorbents.

According to the equations, sorbent mass (A) using the Shell sorbent and extraction time (B) using the Composite sorbent exhibit the highest positive coefficients, while desorption solvent volume (C) exhibits the highest negative coefficient for both sorbents. Therefore, changes in these factors have the most pronounced positive or negative effects on the ER% of the OPPs, respectively. Based on the model, the optimum sorbent mass, extraction time, and desorption solvent volume were predicted to be 45 mg, 11.5 min, and 140 μL for the Shell sorbent and 46 mg, 11.5 min, and 140 μL for the Composite sorbent, respectively (Table S8). The predicted ER% for the optimum conditions was 97.37 % and 98.46 % for the Shell and Composite sorbents, respectively.

### 3.6 Sorbent reusability

Sorbent reusability is a critical factor in evaluating the economic and environmental viability of the D-μ-SPE method. A reusable sorbent reduces waste generation, lowers operational costs, and contributes to a more sustainable analytical workflow. To assess the reusability of the the Shell and Composite sorbents for the extraction of OPPs, a series of consecutive extraction-desorption cycles was performed using the same batch of sorbent. After each cycle, the ER% was determined. The results, summarized in Table S9, demonstrate that the Shell sorbent maintains acceptable performance for at least three extraction-desorption cycles, exhibiting an ER% of 89.67 ± 3.31 % in the third cycle. In contrast, the Composite sorbent shows superior reusability, maintaining consistent extraction efficiency for at least five cycles, with an ER% of 90.82 ± 3.01 % in the fifth cycle. A subsequent decline in ER% was observed in the sixth cycle for both sorbents, suggesting a gradual reduction in the number of active sites or a change in the sorbent's affinity for OPPs over extended use. The higher reusability of the Composite sorbent might be attributed to the protective effect of the MIL-88B support, which enhances the stability and structural integrity of the active Bimetallic Fe/Co-MIL-88A component during repeated extraction and desorption processes. A comprehensive evaluation of both the Shell and composite sorbents was conducted. While their maximum extraction efficiencies under optimized conditions were comparable, as demonstrated in Section 3.2, the composite sorbent was ultimately selected for the application studies due to its superior operational performance. Specifically, it exhibited exceptional reusability and chemical stability over multiple extraction-desorption cycles. The composite's hierarchical structure provides enhanced mechanical and chemical stability that protects the active bimetallic sites from degradation in complex matrices, ensuring consistent, reliable performance. This affirms the practical benefit of the MOF-on-MOF design strategy for extracting OPPs.

### 3.7 Method validation

Table 5 summarizes the method validation results for the determination of selected OPPs using the developed D-μ-SPE method with the Shell and Composite sorbents. The validation parameters assessed include the Linear Dynamic Range (LDR), Coefficient of Determination (R^2^), Relative Standard Deviation (RSD%), Limit of Detection (LOD), Limit of Quantitation (LOQ), and Preconcentration Factor (PF). The LDR was determined by analyzing a series of standard solutions of each OPP at multiple concentration levels. As shown in Table 5, the LDRs spanned from 0.13 to 850 ng mL^−1^ to 0.07–900 ng mL^−1^, for the different analytes, using the Shell and Composite sorbents, respectively. Coefficient of Determination (R^2^) was consistently high, with values ranging from 0.9943 to 0.9974. This indicates a strong linear relationship between the analyte concentration and the analytical signal for all OPPs and both sorbents. The RSD% quantifies the precision or reproducibility of the method. It was calculated by performing multiple (*n* = 3) replicate analyses of a standard solution at a specific concentration level of 5.0 ng mL^−1^. The RSDs ranged from 3.16 % to 4.35 %, demonstrating acceptable precision for all analytes and both sorbents. The LOD was estimated based on the signal-to-noise ratio (S/N) approach, where the LOD was calculated as 3 times the standard deviation of the blank divided by the slope of the calibration curve (LOD = 3σ/S). As shown in Table 5, the LODs range from 0.04 to 0.09 ng mL^−1^ for the Shell sorbent and from 0.02 to 0.08 ng mL^−1^ for the Composite sorbent, suggesting high sensitivity for the proposed method. The LOQ was estimated based on the signal-to-noise ratio (S/N) approach, where the LOQ was calculated as 10 times the standard deviation of the blank divided by the slope of the calibration curve (LOQ = 10σ/S). The LOQs ranged from 0.13 to 0.30 ng mL^−1^ for the Shell sorbent and from 0.07 to 0.27 ng mL^−1^ for the Composite sorbent. In accordance with EU guidelines, the practical method LOQ—the lowest concentration in the original sample that can be quantified with acceptable accuracy and precision—was determined based on the recovery experiments in real matrices (Section 3.8). The lowest validated spike level was 2.0 ng mL^−1^ in the final extract. Given an initial sample mass of 5.0 g and a final extract volume of 10.0 mL, this corresponds to a practical LOQ of 4.0 ng/g for all analytes in solid samples. This value has been added to Table 5 as the Practical LOQ. Preconcentration Factor (PF) was calculated based on the ratio of sample volume to the final volume of solvent (100 μL of ethyl acetate) before injecting into the GC–MS. The PFs were 120 and 190 for determining OPPs using the Shell and Composite sorbents, respectively. Enrichment factor (EF) is calculated from the ratio of the concentration of OPPs in the sample solution to their concentration in the final solvent after the extraction procedure. As demonstrated in Table 5, the EFs were very high, ranging from 46 to 59 for the Shell sorbent and from 52 to 67 for the Composite sorbent, indicating the high enrichment ability of the proposed strategy. In general, the method validation results indicate that the developed D-μ-SPE method, using both sorbents, is a sensitive, precise, and accurate technique for the determination of OPPs.

**Table 5:** The method validation for determining OPPs.

Analytes	Sorbent	LDR^1^ (ngmL^−1^)	R^2^	RSD%	LOD (ngmL^−1^)	LOQ (ngmL^−1^)	Practical LOQ (ngg^−1^)	EF^2^
Chlorpyrifos	Shell sorbent	0.13–850	0.9965	3.47	0.04	0.13	4	51
	Composite sorbent	0.07–900	0.9974	3.29	0.02	0.07	4	59

Phosalone	Shell sorbent	0.30–800	0.9956	4.35	0.09	0.30	4	56
	Composite sorbent	0.27–900	0.9963	4.18	0.08	0.27	4	61
Fenitrothion	Shell sorbent	0.30–850	0.9943	4.28	0.09	0.30	4	46
	Composite sorbent	0.24–900	0.9959	4.25	0.07	0.24	4	55
Profenofos	Shell sorbent	0.27–800	0.9952	3.16	0.08	0.27	4	59
	Composite sorbent	0.17–900	0.9957	3.27	0.05	0.17	4	64

1 Linear dynamic range.

2 Enrichment factor.

### 3.8 Real sample analysis

To evaluate the applicability of the developed method for OPP analysis, a range of representative real samples was procured from a local supermarket in Mashhad, Iran. These included lettuce, tomato, cucumber, commercially produced orange juice, and white grape juice (Sunich, Alifard Co., Tehran, Iran). The sample preparation procedures for each matrix are detailed below.

For the solid vegetable samples, a representative 5.0 g portion of each was finely chopped using a blender to create a homogenized mixture. Acetonitrile (10.0 mL) was then added to this mixture, and the resulting suspension was subjected to mechanical agitation at 200 rpm for 10 min at ambient temperature. This step facilitates the extraction of OPP residues from the vegetable matrix into the acetonitrile solvent. Subsequently, sodium chloride (NaCl, 0.5 g) and anhydrous magnesium sulfate (MgSO_4_, 2.0 g) were added to the mixture. The addition of NaCl promotes phase separation via the salting-out effect, while MgSO_4_ acts as a drying agent to remove residual water from the acetonitrile phase. The mixture was again agitated at 200 rpm for 5 min at room temperature, followed by centrifugation at 5000 rpm for 4 min to separate the supernatant containing the extracted OPPs. The supernatant was then filtered through a 0.45 μm Whatman filter paper to remove any remaining particulate matter. To concentrate the extracted OPPs, the filtrate was evaporated at 30 °C under a gentle stream of nitrogen to a final volume of 1.0 ± 0.3 mL. Finally, the concentrated extract was either diluted to 10.0 mL with distilled water for direct analysis, or spiked with a standard solution of OPPs to a final concentration of 2.0 ng mL^−1^ or 20.0 ng mL^−1^ with a optimum volume for the Shell or Composite sorbents to assess the method's recovery efficiency(Ghorbani et al., 2021b).

The liquid fruit juice samples (10.0 mL each) were initially centrifuged at 4000 rpm for 5 min to remove any suspended particulate matter. The supernatant was then carefully decanted and filtered to ensure the complete removal of solid particles. Similar to the vegetable samples, the filtered supernatant was then either diluted to 10.0 mL with distilled water for direct analysis, or spiked with a standard solution of OPPs to a final concentration of 2.0 ng mL^−1^ or 20.0 ng mL^−1^ with a optimum volume for the Shell or Composite sorbents to assess the method's recovery efficiency.

All prepared real samples, both unspiked and spiked, were analyzed to determine the presence and concentration of OPPs using the optimized method. Triplicate analyses of each sample were performed under identical conditions to enable the determination of recovery rates and standard deviations. The method exhibited acceptable recoveries, ranging from 93.5 % to 103.6 %, and relative standard deviations (RSDs) ranging from 4.37 % to 6.33 % for both sorbents (Table 3).

**Fig. 4:**
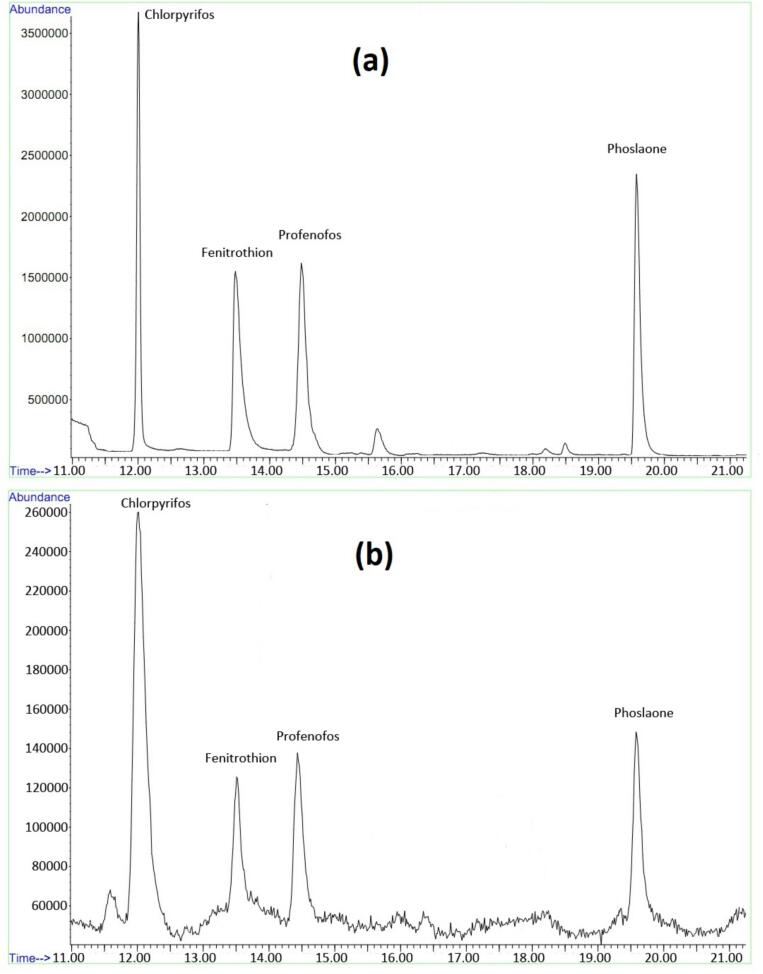
Typical chromatogram of OPPs with a concentration of 20 ngmL^−1^ in a distilled water sample (a) and spiked in an orange juice sample (b) using the Composite sorbent.

The analysis of unspiked real samples revealed that the concentration of OPPs in the majority of samples was below the method's limit of quantitation (LOQ). However, certain OPPs were detected in the vegetable samples, specifically Chlorpyrifos in lettuce and cucumber, and Fenitrothion in Tomato. Notably, the measured concentrations of these OPPs were significantly lower than the established maximum residue levels (MRLs) for those compounds in the respective vegetables. The MRLs are 0.050 mg/Kg for Chlorpyrifos in lettuce and cucumber and 0.50 mg/Kg for Fenitrothion in vegetables (Bempah, Asomaning, & Boateng, 2012). A typical chromatogram of OPPs in distilled water and orange juice samples using the composite sorbent are presented in Fig. 4a and b, respectively.

To accurately quantify the results and evaluate the purification efficiency of the method, the matrix effect (ME) was rigorously assessed. This was achieved by comparing the peak area of the extracted blank samples (Ps) to the peak area of real samples (PR) spiked with the same concentration of analyte (20 ng mL^−1^) based on the following equation:

(4) $$\mathrm{ME}=\frac{P_{R}}{P_{s}}\times 100$$

A ME value of 100 % indicates no effect, while values below or above signify suppression or enhancement, respectively. The results, detailed in Table S10, showed ME values ranging from 87 % to 109 %, indicating mostly soft to moderate matrix effects. The data reveal a clear trend: the Fe/Co-MIL-88A-on-MIL-88B composite sorbent consistently yielded ME values closer to 100 % compared to the Fe/Co-MIL-88A sorbent alone. For instance, in lettuce, the composite reduced the suppression effect for Chlorpyrifos from 90.2 % to 92.5 %. This trend was most pronounced in complex vegetable matrices like tomato, where the composite sorbent significantly mitigated the strong enhancement effect on Fenitrothion, bringing the ME from 107 % down to a more acceptable 107.2 %. The fruit juice samples, being less complex, exhibited milder suppression (87–93 %), which was also more effectively counteracted by the composite sorbent. This systematic reduction in matrix interference demonstrates that the hierarchical MOF-on-MOF structure provides a superior clean-up function, more effectively isolating the analytes from co-extracted matrix components and thereby minimizing ion suppression/enhancement in the GC–MS source.

### 3.9 Comparative performance of shell versus composite sorbent

This comprehensive comparative study reveals the distinct advantages and trade-offs between the Shell (Bimetallic Fe/Co-MIL-88A) and Composite (Bimetallic Fe/Co-MIL-88A-on-MIL-88B) sorbents for OPP extraction. An assessment of method validation parameters (Table 5) indicated that the Composite sorbent generally exhibited a broader LDR, with lower minimum values, than the Shell sorbent. This trend suggests a greater sensitivity for the Composite sorbent at low analyte concentrations. The LODs and LOQs were consistently lower for the Composite sorbent compared to the Shell sorbent. For example, the LOD for Chlorpyrifos was 0.04 ng mL^−1^ for the Shell sorbent and 0.02 ng mL^−1^ for the Composite sorbent. The LOD values for phosalone and fenitrothion were approximately the same for each sorbent (0.09 and 0.07, respectively), and only for profenofos, the LOD changed slightly between the two materials (0.08 and 0.05 ng mL^−1^, respectively). These LODs indicate a superior sensitivity of the Composite sorbent for OPP detection. The Preconcentration Factors were, in general, better for the Composite sorbent. This result reinforces the high affinity of this material towards OPPs. The most significant operational difference was observed in reusability. The reusability of the Shell sorbent was acceptable for three extraction-desorption cycles. In contrast, the Composite sorbent showed superior reusability and retained acceptable performance for at least five cycles (Table S9). A decline in ER% was observed after the fifth cycle. This stark contrast in durability indicates that the Composite sorbent is economically more viable for routine application.

The recoveries are pretty much the same for both sorbents in real samples, so this is not a key point for the material choice (Table 6). Therefore, the critical choice between sorbents hinges on the application's priorities. The Composite sorbent generally outperformed the Shell sorbent in terms of method sensitivity and reusability. The improved analytical performance is likely attributable to the enhanced stability and larger surface area of the composite material. However, the Shell sorbent presents a more cost-effective alternative to the Composite sorbent, as its synthesis requires fewer steps, shorter processing times, and reduced material expenditure. This comparative analysis concludes that while both materials are highly effective, the Composite sorbent is the superior choice for laboratories prioritizing method robustness and long-term cost-effectiveness, whereas the Shell sorbent remains a competent and more economical option.

**Table 6:** Analysis of real vegetable and fruit juice samples for determining OPPs.

Sample	Sorbent	Spiked value (ng mL^−1^)		Chlorpyrifos	Phosalone	Fenitrothion	Profenofos
Lettuce	Shell sorbent	0	Found^1^,^2^	1.42 ± 0.09	ND	ND	ND
			Recovery (%)	–	–	–	–
	Composite sorbent		Found	1.49 ± 0.08	ND	ND	ND
			Recovery (%)	–	–	–	–
	Shell sorbent	2	Found	3.30 ± 0.15	1.87 ± 0.11	1.88 ± 0.11	1.87 ± 0.11
			Recovery (%)	94.0	93.5	94.0	93.5
	Composite sorbent		Found	3.38 ± 0.16	1.89 ± 0.11	1.87 ± 0.10	1.87 ± 0.10
			Recovery (%)	94.5	94.5	93.5	93.5
	Shell sorbent	20	Found	21.92 ± 0.98	19.15 ± 0.89	19.10 ± 0.88	19.05 ± 0.89
			Recovery (%)	102.5	95.8	95.5	95.3
	Composite sorbent		Found	20.63 ± 0.97	19.17 ± 0.87	19.21 ± 0.88	19.18 ± 0.87
			Recovery (%)	95.7	95.9	96.0	95.9

Tomato	Shell sorbent	0	Found	ND	ND	1.29 ± 0.08	ND
			Recovery (%)	–	–	–	–
	Composite sorbent		Found	ND	ND	1.27 ± 0.08	ND
			Recovery (%)	–	–	–	–
	Shell sorbent	2	Found	1.88 ± 0.10	1.87 ± 0.11	3.19 ± 0.15	1.89 ± 0.11
			Recovery (%)	94.0	93.5	95.0	94.5
	Composite sorbent		Found	1.91 ± 0.11	1.87 ± 0.09	3.18 ± 0.15	1.88 ± 0.10
			Recovery (%)	95.0	93.5	95.5	94.0
	Shell sorbent	20	Found	19.18 ± 0.89	19.12 ± 0.90	20.48 ± 0.96	19.08 ± 0.89
			Recovery (%)	95.9	95.6	96.0	95.4
	Composite sorbent		Found	19.18 ± 0.88	19.19 ± 0.89	20.51 ± 0.97	19.20 ± 0.86
			Recovery (%)	95.9	96.0	96.2	96.0

Cucumber	Shell sorbent	0	Found	ND	ND	1.37 ± 0.09	ND
			Recovery (%)	–	–	–	–
	Composite sorbent		Found	ND	ND	1.38 ± 0.08	ND
			Recovery (%)	–	–	–	–
	Shell sorbent	2	Found	1.89 ± 0.11	1.89 ± 0.10	3.28 ± 0.15	1.90 ± 0.11
			Recovery (%)	94.5	94.5	95.0	95.0
	Composite sorbent		Found	1.93 ± 0.11	1.91 ± 0.11	3.31 ± 0.16	1.92 ± 0.11
			Recovery (%)	96.5	95.5	96.5	96.0
	Shell sorbent	20	Found	19.16 ± 0.89	19.21 ± 0.92	22.08 ± 0.98	19.13 ± 0.90
			Recovery (%)	95.8	96.1	103.6	95.6
	Composite sorbent		Found	19.43 ± 0.87	19.41 ± 0.90	20.85 ± 0.94	19.33 ± 0.89
			Recovery (%)	97.2	97.0	97.4	96.6

White grape juice	Shell sorbent	0	Found	ND	ND	ND	ND
			Recovery (%)	–	–	–	–
	Composite sorbent		Found	ND	ND	ND	ND
			Recovery (%)	–	–	–	–
	Shell sorbent	2	Found	1.91 ± 0.12	1.90 ± 0.11	1.89 ± 0.11	1.90 ± 0.10
			Recovery (%)	95.5	95.0	94.5	95.0
	Composite sorbent		Found	1.92 ± 0.10	1.92 ± 0.11	1.91 ± 0.10	1.91 ± 0.10
			Recovery (%)	96.0	96.0	95.5	95.5
	Shell sorbent	20	Found	19.19 ± 0.91	19.18 ± 0.90	19.18 ± 0.89	19.20 ± 0.91
			Recovery (%)	96.0	95.9	95.9	96.0
	Composite sorbent		Found	19.39 ± 0.88	19.41 ± 0.89	19.40 ± 0.88	19.44 ± 0.89
			Recovery (%)	97.0	97.0	97.0	97.2

Orange juice	Shell sorbent	0	Found	ND	ND	ND	ND
			Recovery (%)	–	–	–	–
	Composite sorbent		Found	ND	ND	ND	ND
			Recovery (%)	–	–	–	–
	Shell sorbent	2	Found	1.90 ± 0.10	1.92 ± 0.10	1.92 ± 0.11	1.93 ± 0.10
			Recovery (%)	95.0	96.0	96.0	96.5
	Composite sorbent		Found	1.91 ± 0.10	1.95 ± 0.11	1.92 ± 0.11	1.94 ± 0.10
			Recovery (%)	95.5	97.5	96.0	97.0
	Shell sorbent	20	Found	19.25 ± 0.92	19.26 ± 0.90	19.24 ± 0.91	19.29 ± 0.89
			Recovery (%)	96.2	96.3	96.2	96.4
	Composite sorbent		Found	19.42 ± 0.90	19.43 ± 0.87	19.46 ± 0.85	19.45 ± 0.88
			Recovery (%)	97.1	97.1	97.3	97.2

1 ng mL^−1^.

2 Not detect.

### 3.10 Comparison with other methods

The analytical performance of the developed D-μ-SPE method for OPP determination was benchmarked against previously reported methods. Table S11 summarizes key analytical figures of merit, including the Detection Limit Range (DLR), Limit of Detection (LOD), Relative Standard Deviation (RSD%), and Recovery, for the developed method and several comparable techniques. Compared to other methods, the developed D-μ-SPE method exhibits a compelling combination of advantages. The LODs achieved by the developed method (0.02–0.09 ng mL^−1^) are competitive with or lower than those reported for several other methods, particularly those employing GC-FID [1, 5] or GC-IMS [3]. This indicates a high sensitivity for the determination of OPPs. Also, the method's detection linear range DLR (0.07–900 ng mL^−1^) is generally broader than other methods, particularly compared with SPME, which has a limited DLR of 0.04–20. This broad DLR allows for the accurate determination of a wider range of OPP concentrations without requiring sample dilution. This is only outperformed by the method described by reference 8 with 0.5–2000 ng mL^−1^. The developed method demonstrated satisfactory recoveries (93.7–103.6 %) within the accepted range for analytical methods, showing the proposed strategy extracts most of the analyte. The RSDs obtained (3.16–4.35 %) fall within an acceptable range, indicating good precision and reproducibility of the method. The developed D-μ-SPE method offers a sensitive, precise, and accurate approach for the determination of OPPs in real samples. The developed method's competitive limits of detection, wide linear dynamic range, acceptable recoveries, and satisfactory precision demonstrate its suitability for the effective monitoring of OPPs in vegetable and fruit juice samples, offering a compelling alternative to existing analytical techniques.

## 4 Conclusion

This study presents a comprehensive comparative evaluation of two novel sorbents for the monitoring of OPPs. A streamlined and efficient GC–MS method, coupled with D-μ-SPE, was developed for the quantification of OPPs in diverse vegetable and commercial fruit juice matrices. The two sorbents, termed the Shell Sorbent (Bimetallic Fe/Co-MIL-88A) and the Composite Sorbent (Bimetallic Fe/Co-MIL-88A-on-MIL-88B), were synthesized using solvothermal and hydrothermal procedures. Initial comparative analyses, employing ANOVA and Tukey's Honestly Significant Difference (HSD) test, revealed statistically significant differences in the extraction efficiencies of the Shell Sorbent, MIL-88B, and the Composite Sorbent, indicating a superior affinity of both the Shell and Composite Sorbents for OPPs. A design of experiments (DoE) approach was implemented to optimize key parameters influencing OPP extraction for each sorbent individually. Under optimized conditions, the performance of the developed method was evaluated by analyzing real samples, demonstrating acceptable recoveries (93.5–103.6 %) and precision (RSDs 4.37–6.33 %) for both sorbents. The most decisive finding of this comparative study was the difference in operational robustness. Reusability studies demonstrated that the Shell Sorbent maintained acceptable performance for three extraction-desorption cycles, while the Composite Sorbent exhibited superior reusability for at least five cycles. A comprehensive performance comparison revealed that the Composite Sorbent provided enhanced reusability, lower LODs, and improved linear ranges, EFs, and RSDs for OPP extraction compared to the Shell Sorbent. This direct comparison reveals a clear trade-off between performance and synthesis complexity. While exhibiting a slightly lower analytical sensitivity, the Shell Sorbent presents a more cost-effective alternative to the Composite Sorbent due to its simpler synthesis. However, for applications requiring maximum sensitivity, robustness, and long-term cost-effectiveness through reusability, the Composite Sorbent is the unequivocally superior choice. This work not only provides a highly sensitive analytical method but also delivers a critical evaluation of sorbent selection for routine monitoring of OPPs.

## CRediT authorship contribution statement

**Mahdi Ghorbani:** Writing – review & editing, Writing – original draft, Methodology, Formal analysis, Conceptualization. **Mojgan Ojaghzadeh Khalil Abad:** Writing – original draft, Resources, Data curation. **Majid Keshavarzi:** Writing – original draft, Validation, Investigation.

## Ethical approval

This article lacks research involving human or animal subjects.

## Declaration of competing interest

The authors declare that they have no known competing financial interests or personal relationships that could have appeared to influence the work reported in this paper.

## Data Availability

All data generated or analyzed during this study are included in this published article and its supplementary information files.

## Appendix A Supplementary data

**Supplementary Data for:** A Novel MOF-on-MOF Composite versus its MOF Shell: A Comparative Sorbent Study for Dispersive Micro-Solid Phase Extraction of Pesticides in Food Samples. This file contains: **Table S1:** Normality test results (Shapiro-Wilk) for the extraction of organophosphorus pesticides (OPPs) using the different sorbents. **Table S2:** One-way ANOVA and Tukey's HSD test comparing the extraction efficiency of the three sorbents. **Table S3:** Comparison of desorption solvents for the Shell sorbent using one-way ANOVA and Tukey's HSD test. **Table S4:** Comparison of desorption solvents for the Composite sorbent using one-way ANOVA and Tukey's HSD test. **Table S5:** Analysis of variance (ANOVA) for the screening design experiments evaluating the effect of seven factors on extraction efficiency for both sorbents. **Table S6:** ANOVA for the optimization design (Response Surface Methodology) for both sorbents. **Table S7:** Statistical parameters for the quadratic models obtained during the optimization stage. **Table S8:** Optimum values for the significant factors and the predicted extraction recovery (ER%) under these conditions. Table S9: Sorbent reusability study over six extraction-desorption cycles. **Table S10:** Matrix Effect (ME) evaluation for individual OPPs in different food samples. **Table S11:** Comparison of the developed method with other reported methods for the determination of OPPs. Image 1

## References

[bb0005] Abad M.O.K., Masrournia M., Javid A. (2023). Simultaneous determination of paclitaxel and vinorelbine from environmental water and urine samples based on dispersive micro solid phase extraction-HPLC using a green and novel MOF-on-MOF sorbent composite. Microchemical Journal.

[bb0010] Abad M.O.K., Masrournia M., Javid A. (2024). Synthesis of novel MOF-on-MOF composite as a magnetic sorbent to dispersive micro solid phase extraction of benzodiazepine drugs prior to determination with HPLC-UV. Microchemical Journal.

[bb0015] Alhmaunde A., Masrournia M., Javid A. (2022). Facile synthesis of new magnetic sorbent based on MOF-on-MOF for simultaneous extraction and determination of three benzodiazepines in various environmental water samples using dispersive micro solid-phase extraction and HPLC. Microchemical Journal.

[bb0020] Alizadeh R., Mashalavi B., Yeganeh Faal A., Seidi S. (2021). Development of ultrasound assisted dispersive micro solid phase extraction based on CuO nanoplate-polyaniline composite as a new sorbent for insecticides analysis in wheat samples. Microchemical Journal.

[bb0025] Aroniadou-Anderjaska V., Figueiredo T.H., de Araujo Furtado M., Pidoplichko V.I., Braga M.F. (2023). Mechanisms of organophosphate toxicity and the role of acetylcholinesterase inhibition. Toxics.

[bb0035] Bedair A., Abdelhameed R., Hammad S.F., Abdallah I.A., Mansour F.R. (2024). Applications of metal organic frameworks in dispersive micro solid phase extraction (D-μ-SPE). Journal of Chromatography A.

[bb0040] Bempah C.K., Asomaning J., Boateng J. (2012). Market basket survey for some pesticides residues in fruits and vegetables from Ghana. Journal of Microbiology, Biotechnology and Food Sciences.

[bb0045] Bhattu M., Kathuria D., Billing B.K., Verma M. (2022). Chromatographic techniques for the analysis of organophosphate pesticides with their extraction approach: A review (2015–2020). Analytical Methods.

[bb0050] Cai G., Yan P., Zhang L., Zhou H.-C., Jiang H.-L. (2021). Metal–organic framework-based hierarchically porous materials: Synthesis and applications. Chemical Reviews.

[bb0055] Castañeda F.N., Prince D.L., Peirano S.R., Giovannoni S., Echevarría R.N., Keunchkarian S., Reta M. (2024). New sorbents for sample pretreatment: Development and applications. TrAC Trends in Analytical Chemistry.

[bb0060] Chen Y., Yang Z., Nian B., Yu C., Maimaiti D., Chai M., Xu D. (2024). Mechanisms of neurotoxicity of organophosphate pesticides and their relation to neurological disorders. Neuropsychiatric Disease and Treatment.

[bb0065] Darvishnejad F., Raoof J.B., Ghani M., Ojani R. (2023). Keggin-type polyoxometalate embedded polyvinylidene fluoride for thin film microextraction of organophosphorus pesticides. Food Chemistry: X.

[bb0070] D’Orazio G. (2024). Microextraction techniques: Fundamentals, applications and recent developments.

[bb0075] Dozein S.V., Masrournia M., Es’haghi Z., Bozorgmehr M.R. (2021). Development of a new magnetic dispersive solid-phase microextraction coupled with GC-MS for the determination of five organophosphorus pesticides from vegetable samples. Food Analytical Methods.

[bb0080] Dugheri S., Marrubini G., Mucci N., Cappelli G., Bonari A., Pompilio I., Arcangeli G. (2021). A review of micro-solid-phase extraction techniques and devices applied in sample pretreatment coupled with chromatographic analysis. Acta Chromatographica.

[bb0085] El-Deen A.K. (2024). An overview of recent advances and applications of matrix solid-phase dispersion. Separation & Purification Reviews.

[bb0090] Fu H., Tan P., Wang R., Li S., Liu H., Yang Y., Wu Z. (2022). Advances in organophosphorus pesticides pollution: Current status and challenges in ecotoxicological, sustainable agriculture, and degradation strategies. Journal of Hazardous Materials.

[bb0095] Ganie S.Y., Javaid D., Hajam Y.A., Reshi M.S. (2022). Mechanisms and treatment strategies of organophosphate pesticide induced neurotoxicity in humans: A critical appraisal. Toxicology.

[bb0100] Ghorbani M., Keshavarzi M., Pakseresht M., Mohammadi P., Abad M.O.K., Mehraban A. (2024). Advancements, applications, and prospects of metal-organic frameworks and their derivatives as distinct sorbents in exhaustive and non-exhaustive extraction strategies. Microchemical Journal.

[bb0105] Ghorbani M., Mohammadi P., Keshavarzi M., Saghi M.H., Mohammadi M., Shams A., Aghamohammadhasan M. (2021). Simultaneous determination of organophosphorus pesticides residues in vegetable, fruit juice, and milk samples with magnetic dispersive micro solid-phase extraction and chromatographic method; recruitment of simplex lattice mixture design for optimization of novel sorbent composites. Analytica Chimica Acta.

[bb0110] Gumus Z.P., Soylak M. (2025). Tailoring a MOF-on-MOF (ZIF-8-on-NH2-MIL-53 (Al)) hybrid composite for micro solid phase extraction of furosemide in artificial biological fluids prior to LC-q-TOF/MS detection. Advances in Sample Preparation.

[bb0115] Haldar R., Wöll C. (2021). Hierarchical assemblies of molecular frameworks—MOF-on-MOF epitaxial heterostructures. Nano Research.

[bb0120] Hashemi S.H., Kaykhaii M. (2024). Porous polymer sorbents in micro solid phase extraction: Applications, advantages, and challenges. Topics in Current Chemistry.

[bb0125] Ige O.E., Aliu F.P., Omole A.E., Alabi O.O. (2025). The interplay of pesticides and climate change: Environmental dynamics and challenges.

[bb0130] Jagirani M.S., Soylak M. (2024). Green sorbents for the solid phase extraction of trace species. Current Opinion in Green and Sustainable Chemistry.

[bb0135] Jaiswal S., Singh B., Dhingra I., Joshi A., Kodgire P. (2024). Bioremediation and bioscavenging for elimination of organophosphorus threats: An approach using enzymatic advancements. Environmental Research.

[bb0140] Ke F., Luo G., Chen P., Jiang J., Yuan Q., Cai H., Wan X. (2016). Porous metal–organic frameworks adsorbents as a potential platform for defluoridation of water. Journal of Porous Materials.

[bb0145] Korrani Z.S., Khalili E., Kamyab H., Ibrahim W.A.W., Hashim H. (2023). A new solid phase extraction sorbent developed based on cyanopropyl functionalized silica nanoparticles for organophosphorus pesticides determination. Environmental Research.

[bb0150] Liu Y., Wang Z., Liu M., Zhang T., Liu S., Lu K. (2025). Characterization, sources and risk assessment of organochlorine pesticides (OCPs), organophosphorus pesticides (OPPs) and polycyclic aromatic hydrocarbons (PAHs) in agricultural soils from Jilin Province in Northeast China. Stochastic Environmental Research and Risk Assessment.

[bb0155] Mane P.V., Rego R.M., Yap P.L., Losic D., Kurkuri M.D. (2024). Unveiling cutting-edge advances in high surface area porous materials for the efficient removal of toxic metal ions from water. Progress in Materials Science.

[bb0160] Naz S., Iqbal S., Manan A., Chatha M., Zia M. (2023). The web of life: Role of pesticides in the biodiversity decline. International Journal of Forest Sciences.

[bb0165] Pan Y., Liu X., Liu J., Wang J., Liu J., Gao Y., Ma N. (2022). Determination of organophosphorus in dairy products by graphitic carbon nitride combined molecularly imprinted microspheres with ultra performance liquid chromatography. Food Chemistry: X.

[bb0170] Patra K., Dey S., Solanki C., Sengupta A., Mittal V.K. (2025). Harnessing advanced porous materials, covalent organic frameworks, and porous organic polymers as next-generation porous frameworks for targeted removal of emerging water contaminants. ACS Applied Engineering Materials.

[bb0175] Qin P., Zhu S., Mu M., Gao Y., Cai Z., Lu M. (2023). Constructing cactus-like mixed dimensional MOF@ MOF as sorbent for extraction of bisphenols from environmental water. Chinese Chemical Letters.

[bb0180] Rabeie B., Mahmoodi N.M. (2024). Heterogeneous MIL-88A on MIL-88B hybrid: A promising eco-friendly hybrid from green synthesis to dual application (adsorption and photocatalysis) in tetracycline and dyes removal. Journal of Colloid and Interface Science.

[bb0185] Radowan A.A.A. (2024). Analytical techniques for determining pesticide residues in food: A comprehensive review. International Journal of Materials Technology and Innovation.

[bb0190] Sanyal D., Mathur P. (2022). Advanced adsorbent mediated extraction techniques for the separation of antibiotics from food, biological, and environmental matrices. Separation & Purification Reviews.

[bb0195] Sarailoo M., Afshari S., Asghariazar V., Safarzadeh E., Dadkhah M. (2022). Cognitive impairment and neurodegenerative diseases development associated with organophosphate pesticides exposure: A review study. Neurotoxicity Research.

[bb0200] Sridhar G.R. (2021). Acetylcholinesterase inhibitors (galantamine, rivastigmine, and donepezil). NeuroPsychopharmacotherapy.

[bb0205] Veloo K.V., Ibrahim N.A.S. (2021). Analytical extraction methods and sorbents’ development for simultaneous determination of organophosphorus pesticides’ residues in food and water samples: A review. Molecules.

[bb0210] Zhang M.Y., Li J.K., Wang R., Zhao S.N., Zang S.Q., Mak T.C. (2021). Construction of core–shell MOF@ COF hybrids with controllable morphology adjustment of COF shell as a novel platform for photocatalytic cascade reactions. Advanced Science.

[bb0215] Zhang W., Giesy J.P., Wang P. (2022). Organophosphate esters in agro-foods: Occurrence, sources and emerging challenges. Science of the Total Environment.

[bb0220] Zorainy M.Y., Kaliaguine S., Gobara M., Elbasuney S., Boffito D.C. (2022). Microwave-assisted synthesis of the flexible iron-based MIL-88B metal–organic framework for advanced energetic systems. Journal of Inorganic and Organometallic Polymers and Materials.

