# Supplementary material for: A novel MOF-on-MOF composite versus its MOF shell: a comparative sorbent study for dispersive micro-solid phase extraction of pesticides in food samples
Source: Food Chem X. 2025 Sep 27;31:103091. doi: 10.1016/j.fochx.2025.103091 (PMC12513179; doi:10.1016/j.fochx.2025.103091)
Supplement: Supplementary file 1 — Supplementary Data for: A Novel MOF-on-MOF Composite versus its MOF Shell: A Comparative Sorbent Study for Dispersive Micro-Solid Phase Extraction of Pesticides in Food Samples. This file contains: Table S1: Normality test results (Shapiro-Wilk) for the extraction of organophosphorus pesticides (OPPs) using the different sorbents. Table S2: One-way ANOVA and Tukey's HSD test comparing the extraction efficiency of the three sorbents. Table S3: Comparison of desorption solvents for the Shell sorbent using one-way ANOVA and Tukey's HSD test. Table S4: Comparison of desorption solvents for the Composite sorbent using one-way ANOVA and Tukey's HSD test. Table S5: Analysis of variance (ANOVA) for the screening design experiments evaluating the effect of seven factors on extraction efficiency for both sorbents. Table S6: ANOVA for the optimization design (Response Surface Methodology) for both sorbents. Table S7: Statistical parameters for the quadratic models obtained during the optimization stage. Table S8: Optimum values for the significant factors and the predicted extraction recovery (ER%) under these conditions. Table S9: Sorbent reusability study over six extraction-desorption cycles. Table S10: Matrix Effect (ME) evaluation for individual OPPs in different food samples. Table S11: Comparison of the developed method with other reported methods for the determination of OPPs. [file mmc1.docx]

**Electronic Supplementary Material**

**A Novel MOF-on-MOF Composite versus its MOF Shell: A Comparative Sorbent Study for Dispersive Micro-Solid Phase Extraction of Pesticides in Food Samples**

Mahdi Ghorbani^1^, Mojgan Ojaghzadeh Khalil Abad^2^, Majid Keshavarzi^3^

^1^ Department of Chemistry, Faculty of Sciences, Ferdowsi University of Mashhad, Mashhad, Iran

**^2^** Department of Chemistry, Mashhad Branch, Islamic Azad University, Mashhad, Iran

^3^ Department of Pharmacodynamics and Toxicology, School of Pharmacy, Mashhad University of Medical Sciences, Mashhad, Iran

* Corresponding author.

E-mail address: [ghorbani267@yahoo.com](mailto:ghorbani267@yahoo.com), ghorbani267@mail.um.ac.ir

Table S1. Test of normality for extracting OPPs using the sorbents

| Sorbent | Shapiro-Wilk | | |
| --- | --- | --- | --- |
|  | Statistic | df | Significance |
| MIL-88B | 0.912 | 3 | 0.426 |
| Shell sorbent | 0.998 | 3 | 0.916 |
| Composite sorbent | 0.963 | 3 | 0.632 |

Table S2. One-way ANOVA and Tukey HSD for comparing the sorbents in the OPP extraction

| **One-way ANOVA** | | | | | |
| --- | --- | --- | --- | --- | --- |
|  | Sum of Squares | df | Mean Square | F | Significance |
| Between groups | 805.677 | 2 | 402.838 | 51.002 | 0.000 |
| Within groups | 47.391 | 6 | 7.899 |  |  |
| Total | 853.068 | 8 |  |  |  |

| **Tukey HSD** | | | | | | |
| --- | --- | --- | --- | --- | --- | --- |
| (I) Sorbent | (J) Sorbent | Mean Difference (I-J) | Std. Error | Significance | 95% Confidence Interval | |
|  |  |  |  |  | Lower Bound | Upper Bound |
| MIL-88B | Shell sorbent | -18.67000^*^ | 2.29470 | 0.000 | -25.7108 | -11.6292 |
|  | Composite sorbent | -21.22667^*^ | 2.29470 | 0.000 | -28.2674 | -14.1859 |
| Shell sorbent | MIL-88B | 18.67000^*^ | 2.29470 | 0.000 | 11.6292 | 25.7108 |
|  | Composite sorbent | -2.55667 | 2.29470 | 0.541 | -9.5974 | 4.4841 |
| Bimetallic Fe/Co-MIL-88A on MIL-88B | MIL-88B | 21.22667^*^ | 2.29470 | 0.000 | 14.1859 | 28.2674 |
|  | Shell sorbent | 2.55667 | 2.29470 | 0.541 | -4.4841 | 9.5974 |

Table S3. One-way ANOVA and Tukey HSD for comparing the desorption solvents using the Shell sorbent

| One-Way ANOVA using the Shell sorbent | | | | | |
| --- | --- | --- | --- | --- | --- |
|  | Sum of Squares | df | Mean Square | F | Significance |
| Between Groups | 368.231 | 5 | 73.646 | 14.257 | 0.000 |
| Within Groups | 61.989 | 12 | 5.166 |  |  |
| Total | 430.219 | 17 |  |  |  |

| Tukey HSD using the Shell sorbent | | | | | | |
| --- | --- | --- | --- | --- | --- | --- |
| (I) Desorption solvent | (J) Desorption solvent | Mean Difference (I-J) | Std. Error | Significance | 95% Confidence Interval | |
|  |  |  |  |  | Lower Bound | Upper Bound |
| Methanol | Ethanol | 1.22000 | 1.85575 | 0.984 | -5.0133 | 7.4533 |
|  | 2-propanol | 2.91333 | 1.85575 | 0.631 | -3.3200 | 9.1467 |
|  | Acetonitrile | -6.40333^*^ | 1.85575 | 0.043 | -12.6367 | -.1700 |
|  | Ethyl acetate | -.01667 | 1.85575 | 1.000 | -6.2500 | 6.2167 |
|  | Acetone | 8.89000^*^ | 1.85575 | 0.005 | 2.6567 | 15.1233 |
| Ethanol | Methanol | -1.22000 | 1.85575 | 0.984 | -7.4533 | 5.0133 |
|  | 2-propanol | 1.69333 | 1.85575 | 0.936 | -4.5400 | 7.9267 |
|  | Acetonitrile | -7.62333^*^ | 1.85575 | 0.014 | -13.8567 | -1.3900 |
|  | Ethyl acetate | -1.23667 | 1.85575 | 0.983 | -7.4700 | 4.9967 |
|  | Acetone | 7.67000^*^ | 1.85575 | 0.014 | 1.4367 | 13.9033 |
| 2-propanol | Methanol | -2.91333 | 1.85575 | 0.631 | -9.1467 | 3.3200 |
|  | Ethanol | -1.69333 | 1.85575 | 0.936 | -7.9267 | 4.5400 |
|  | Acetonitrile | -9.31667^*^ | 1.85575 | 0.003 | -15.5500 | -3.0833 |
|  | Ethyl acetate | -2.93000 | 1.85575 | 0.626 | -9.1633 | 3.3033 |
|  | Acetone | 5.97667 | 1.85575 | 0.063 | -.2567 | 12.2100 |
| Acetonitrile | Methanol | 6.40333^*^ | 1.85575 | 0.043 | .1700 | 12.6367 |
|  | Ethanol | 7.62333^*^ | 1.85575 | 0.014 | 1.3900 | 13.8567 |
|  | 2-propanol | 9.31667^*^ | 1.85575 | 0.003 | 3.0833 | 15.5500 |
|  | Ethyl acetate | 6.38667^*^ | 1.85575 | 0.044 | .1533 | 12.6200 |
|  | Acetone | 15.29333^*^ | 1.85575 | 0.000 | 9.0600 | 21.5267 |
| Ethyl acetate | Methanol | .01667 | 1.85575 | 1.000 | -6.2167 | 6.2500 |
|  | Ethanol | 1.23667 | 1.85575 | 0.983 | -4.9967 | 7.4700 |
|  | 2-propanol | 2.93000 | 1.85575 | 0.626 | -3.3033 | 9.1633 |
|  | Acetonitrile | -6.38667^*^ | 1.85575 | 0.044 | -12.6200 | -.1533 |
|  | Acetone | 8.90667^*^ | 1.85575 | 0.004 | 2.6733 | 15.1400 |
| Acetone | Methanol | -8.89000^*^ | 1.85575 | 0.005 | -15.1233 | -2.6567 |
|  | Ethanol | -7.67000^*^ | 1.85575 | 0.014 | -13.9033 | -1.4367 |
|  | 2-propanol | -5.97667 | 1.85575 | 0.063 | -12.2100 | .2567 |
|  | Acetonitrile | -15.29333^*^ | 1.85575 | 0.000 | -21.5267 | -9.0600 |
|  | Ethyl acetate | -8.90667^*^ | 1.85575 | 0.004 | -15.1400 | -2.6733 |

Table S4. One-way ANOVA and Tukey HSD for comparing the desorption solvents using the Composite sorbent

| One-Way ANOVA using the Composite sorbent | | | | | |
| --- | --- | --- | --- | --- | --- |
|  | Sum of Squares | df | Mean Square | F | Significance |
| Between Groups | 514.082 | 5 | 102.816 | 15.388 | 0.000 |
| Within Groups | 80.180 | 12 | 6.682 |  |  |
| Total | 594.262 | 17 |  |  |  |

| Tukey HSD using the Composite sorbent | | | | | | |
| --- | --- | --- | --- | --- | --- | --- |
| (I) Desorption solvent | (J) Desorption solvent | Mean Difference (I-J) | Std. Error | Significance | 95% Confidence Interval | |
|  |  |  |  |  | Lower Bound | Upper Bound |
| Methanol | Ethanol | 1.75000 | 2.11056 | 0.956 | -5.3392 | 8.8392 |
|  | 2-propanol | 4.03667 | 2.11056 | 0.440 | -3.0525 | 11.1259 |
|  | Acetonitrile | -7.23667^*^ | 2.11056 | 0.044 | -14.3259 | -.1475 |
|  | Ethyl acetate | 3.53667 | 2.11056 | 0.570 | -3.5525 | 10.6259 |
|  | Acetone | 10.69333^*^ | 2.11056 | 0.003 | 3.6041 | 17.7825 |
| Ethanol | Methanol | -1.75000 | 2.11056 | 0.956 | -8.8392 | 5.3392 |
|  | 2-propanol | 2.28667 | 2.11056 | 0.879 | -4.8025 | 9.3759 |
|  | Acetonitrile | -8.98667^*^ | 2.11056 | 0.011 | -16.0759 | -1.8975 |
|  | Ethyl acetate | 1.78667 | 2.11056 | 0.952 | -5.3025 | 8.8759 |
|  | Acetone | 8.94333^*^ | 2.11056 | 0.011 | 1.8541 | 16.0325 |
| 2-propanol | Methanol | -4.03667 | 2.11056 | 0.440 | -11.1259 | 3.0525 |
|  | Ethanol | -2.28667 | 2.11056 | 0.879 | -9.3759 | 4.8025 |
|  | Acetonitrile | -11.27333^*^ | 2.11056 | 0.002 | -18.3625 | -4.1841 |
|  | Ethyl acetate | -.50000 | 2.11056 | 1.000 | -7.5892 | 6.5892 |
|  | Acetone | 6.65667 | 2.11056 | 0.070 | -.4325 | 13.7459 |
| Acetonitrile | Methanol | 7.23667^*^ | 2.11056 | 0.044 | .1475 | 14.3259 |
|  | Ethanol | 8.98667^*^ | 2.11056 | 0.011 | 1.8975 | 16.0759 |
|  | 2-propanol | 11.27333^*^ | 2.11056 | 0.002 | 4.1841 | 18.3625 |
|  | Ethyl acetate | 10.77333^*^ | 2.11056 | 0.003 | 3.6841 | 17.8625 |
|  | Acetone | 17.93000^*^ | 2.11056 | 0.000 | 10.8408 | 25.0192 |
| Ethyl acetate | Methanol | -3.53667 | 2.11056 | 0.570 | -10.6259 | 3.5525 |
|  | Ethanol | -1.78667 | 2.11056 | 0.952 | -8.8759 | 5.3025 |
|  | 2-propanol | .50000 | 2.11056 | 1.000 | -6.5892 | 7.5892 |
|  | Acetonitrile | -10.77333^*^ | 2.11056 | 0.003 | -17.8625 | -3.6841 |
|  | Acetone | 7.15667^*^ | 2.11056 | 0.047 | .0675 | 14.2459 |
| Acetone | Methanol | -10.69333^*^ | 2.11056 | 0.003 | -17.7825 | -3.6041 |
|  | Ethanol | -8.94333^*^ | 2.11056 | 0.011 | -16.0325 | -1.8541 |
|  | 2-propanol | -6.65667 | 2.11056 | 0.070 | -13.7459 | .4325 |
|  | Acetonitrile | -17.93000^*^ | 2.11056 | 0.000 | -25.0192 | -10.8408 |
|  | Ethyl acetate | -7.15667^*^ | 2.11056 | 0.047 | -14.2459 | -.0675 |

Table S5. ANOVA for analyzing of screening design using the Shell or Composite sorbents

| Source | df | Shell sorbent | | | | Composite sorbent | | | | |
| --- | --- | --- | --- | --- | --- | --- | --- | --- | --- | --- |
|  |  | **Sum of Squares** | **Mean Square** | **p-value** | **Significance** | **Sum of Squares** | **Mean Square** | **p-value** | **Significance** |  |
| **Model** | 7 | 5669.31 | 809.90 | < 0.0001 | + | 5367.26 | 766.75 | 0.0001 | + |  |
| A-pH | 1 | 81.84 | 81.84 | 0.1902 | - | 196.80 | 196.80 | 0.0560 | - |  |
| B-Sorbent mass | 1 | 2152.14 | 2152.14 | < 0.0001 | + | 1676.23 | 1676.23 | 0.0001 | + |  |
| C-Sample volume | 1 | 28.12 | 28.12 | 0.4278 | - | 79.87 | 79.87 | 0.1959 | - |  |
| D-Extraction time | 1 | 1963.07 | 1963.07 | < 0.0001 | + | 2208.30 | 2208.30 | < 0.0001 | + |  |
| E-Desorption time | 1 | 100.45 | 100.45 | 0.1510 | - | 161.36 | 161.36 | 0.0784 | - |  |
| F-Desorption solvent volume | 1 | 1327.95 | 1327.95 | 0.0003 | + | 1005.22 | 1005.22 | 0.0008 | + |  |
| G-Salt percentage | 1 | 15.73 | 15.73 | 0.5499 | - | 39.48 | 39.48 | 0.3517 | - |  |
| **Residual** | 9 | 367.01 | 40.78 |  |  | 368.39 | 40.93 |  |  |  |
| **Cor Total** | 16 | 6036.32 |  |  |  | 5735.65 |  |  |  |  |

Table S6. ANOVA for analyzing of optimization design using the Shell or Composite sorbents.

| Source | df | Shell sorbent | | | | Composite sorbent | | | |
| --- | --- | --- | --- | --- | --- | --- | --- | --- | --- |
|  |  | **Sum of Squares** | **Mean Square** | **p-value** | **Significance** | **Sum of Squares** | **Mean Square** | **p-value** | **Significance** |
| **Model** | 9 | 7034.53 | 781.61 | < 0.0001 | + | 6718.45 | 746.49 | < 0.0001 | + |
| A-Sorbent mass | 1 | 1344.44 | 1344.44 | < 0.0001 | + | 880.97 | 880.97 | < 0.0001 | + |
| B-Extraction time | 1 | 1249.92 | 1249.92 | < 0.0001 | + | 1237.66 | 1237.66 | < 0.0001 | + |
| C-Desorption solvent volume | 1 | 804.61 | 804.61 | < 0.0001 | + | 866.95 | 866.95 | < 0.0001 | + |
| AB | 1 | 342.17 | 342.17 | 0.0022 | + | 148.61 | 148.61 | 0.0084 | + |
| AC | 1 | 238.49 | 238.49 | 0.0067 | + | 501.49 | 501.49 | 0.0001 | + |
| BC | 1 | 0.0000 | 0.0000 | 1.0000 | - | 2.95 | 2.95 | 0.6546 | - |
| A² | 1 | 43.65 | 43.65 | 0.1762 | - | 237.36 | 237.36 | 0.0020 | + |
| B² | 1 | 731.46 | 731.46 | 0.0001 | + | 336.72 | 336.72 | 0.0006 | + |
| C² | 1 | 167.70 | 167.70 | 0.0172 | + | 203.18 | 203.18 | 0.0033 | + |
| **Residual** | 10 | 206.02 | 20.60 |  |  | 138.84 | 13.88 |  |  |
| Lack of Fit | 5 | 191.38 | 38.28 | 0.0068 | - | 110.89 | 22.18 | 0.0783 | - |
| Pure Error | 5 | 14.64 | 2.93 |  |  | 27.95 | 5.59 |  |  |
| **Cor Total** | 19 | 7240.56 |  |  |  | 6857.29 |  |  |  |

### Table S7. Statistical parameters for the equations obtained in the optimization stage

| Shell sorbent | | | | |  | Composite sorbent | | | | |
| --- | --- | --- | --- | --- | --- | --- | --- | --- | --- | --- |
| **Std. Dev.** | 4.54 |  | **R²** | 0.9715 |  | **Std. Dev.** | 3.73 |  | **R²** | 0.9798 |
| **Mean** | 74.45 |  | **Adjusted R²** | 0.9459 |  | **Mean** | 76.96 |  | **Adjusted R²** | 0.9615 |
| **C.V. %** | 6.10 |  | **Predicted R²** | 0.8737 |  | **C.V. %** | 4.84 |  | **Predicted R²** | 0.9089 |
|  |  |  | **Adeq Precision** | 20.6815 |  |  |  |  | **Adeq Precision** | 24.1785 |

Table S8. Optimum value of the significant factors and Predicted ER% in the optimization stage

| Factor | Name | Shell sorbent | | Composite sorbent | |
| --- | --- | --- | --- | --- | --- |
|  |  | **Obtained value** | **Rounded value** | **Obtained value** | **Rounded value** |
| A | Sorbent mass | 45.23 | 45 mg | 45.99 | 46 mg |
| B | Extraction time | 11.16 | 11 min | 11.57 | 11.5 min |
| C | Desorption solvent volume | 139.91 | 140 µL | 139.41 | 140 µL |

| Solution 1 of 100 Response | Predicted Mean | Predicted Median | Std Dev | SE Mean | 95% CI low for Mean | 95% CI high for Mean |
| --- | --- | --- | --- | --- | --- | --- |
| ER% for the Shell sorbent | 97.3873 | 97.3873 | 4.53897 | 1.90126 | 93.1511 | 101.624 |
| ER% for the Composite sorbent | 98.1539 | 98.1539 | 3.72607 | 1.94709 | 93.8155 | 102.492 |

Two-sided    Confidence = 95%    Population = 99%

Table S9. The sorbent reusability for extracting OPPs

| Cycle | ER%±SD | |
| --- | --- | --- |
|  | Shell sorbent | **Composite sorbent** |
| 1 | 96.53±2.59 | 97.84±2.47 |
| 2 | 93.41±2.87 | 96.76±2.53 |
| 3 | 89.67±3.31 | 94.93±2.64 |
| 4 | 83.08±3.54 | 92.15±2.81 |
| 5 | 78.91±3.67 | 90.82±3.01 |
| 6 | 72.47±3.88 | 85.54±3.48 |

Table S10. Matrix Effect (ME) evaluation for individual OPPs using different sorbents (mean ± SD, n=3).

| Sample | Sorbent | Chlorpyrifos | Phosalone | Fenitrothion | Profenofos |
| --- | --- | --- | --- | --- | --- |
| Lettuce | Shell sorbent | 90.2 ± 1.8 | 91.5±1.6 | 90.1±1.9 | 89.3±1.7 |
|  | Composite sorbent | 92.5±1.6 | 92.1±1.4 | 91.3±1.8 | 90.6±1.6 |
| Tomato | Shell sorbent | 105.4±2.1 | 91.7±1.9 | 1.07.9±2.3 | 90.8±2.0 |
|  | Composite sorbent | 104.1±1.9 | 92.7±1.8 | 107.2±2.1 | 91.2±1.9 |
| Cucumber | Shell sorbent | 89.4±1.7 | 90.2±1.6 | 105.8±2.2 | 91.3±1.7 |
|  | Composite sorbent | 90.1±1.6 | 90.5±1.4 | 103.2±1.9 | 92.1±1.6 |
| White grape juice | Shell sorbent | 91.3±1.5 | 90.8±1.6 | 91.2±1.8 | 91.4±1.5 |
|  | Composite sorbent | 91.9±1.4 | 91.2±1.6 | 91.7±1.7 | 92.1±1.6 |
| Orange juice | Shell sorbent | 92.1±1.5 | 90.3±1.7 | 92.3±1.6 | 92.4±1.6 |
|  | Composite sorbent | 92.8±1.5 | 91.7±1.5 | 92.9±1.5 | 93.2±1.4 |

Table S11. The comparison of the method with other methods for the determination of OPPs.

| Date | Extraction | Detection | DLR  (ng mL^-1^) | LOD  (ng mL^-1^) | RSD% | Recovery | Ref |
| --- | --- | --- | --- | --- | --- | --- | --- |
| 2018 | MSPE^4^ | GC-FID | 0.3-500 | 0.1-0.3 | 4.7-8.1 | 88.1-99.2 | [1] |
| 2018 | IL-DLLME^5^ | GC-MS | ---- | 5-16 | 4.1-9.7 | 85-118 | [2] |
| 2019 | MDSPME^6^ | GC-IMS^7^ | 2-1000 | 0.46-1.0 | 3-9 | >82 | [3] |
| 2020 | DSPME^8^ | GC-MS | 0.2-1300 | 0.06-1.49 | 4.12-4.73 | 93.9-106.7 | [4] |
| 2021 | dµSPE | GC-FID | 0.11-1200 | 0.03-0.11 | 4.06-4.59 | 83.7-98.1 | [5] |
| 2022 | SPE | GC-MS | 0.5-500 | 0.32-0.82 | 2.1-7.2 | 91.2-103.7 | [6] |
| 2023 | SPME | GC-MS | 0.04-20 | 0.012-0.051 | 3.2-8.1 | 92.0-103.8 | [7] |
| 2024 | SPME | GC | 0.5-2000 | 0.25-0.50 | < 7% | 93-117 | [8] |
| ---- | dµSPE | GC-MS | 0.07-900 | 0.02-0.09 | 3.16-4.35 | 93.7-103.6 | This work |

^1^Magnetic solid-phase extraction, ^2^Ionic liquid-dispersive liquid-liquid microextraction, ^3^Magnetic dispersive solid-phase microextractions, ^4^Gas chromatography- Ion mobility spectrometry detection, ^5^Dispersive solid-phase microextraction

**References**

[1] A. Targhoo, A. Amiri, M. Baghayeri (2018) Magnetic nanoparticles coated with poly (p-phenylenediamine-co-thiophene) as a sorbent for preconcentration of organophosphorus pesticides. Microchimica Acta 185 15.

[2] J. Cacho, N. Campillo, P. Viñas, M. Hernández-Córdoba (2018) In situ ionic liquid dispersive liquid-liquid microextraction coupled to gas chromatography-mass spectrometry for the determination of organophosphorus pesticides. Journal of Chromatography A 1559 95-101.

[3] M. Kermani, M.T. Jafari, M. Saraji (2019) Porous magnetized carbon sheet nanocomposites for dispersive solid-phase microextraction of organophosphorus pesticides prior to analysis by gas chromatography-ion mobility spectrometry. Microchimica Acta 186 88.

[4] S.V. Dozein, M. Masrournia, Z. Es’haghi, M.R. Bozorgmehr (2020) Development of a New Magnetic Dispersive Solid-Phase Microextraction Coupled with GC-MS for the Determination of Five Organophosphorus Pesticides from Vegetable Samples. Food Analytical Methods 1-13.

[5] M. Ghorbani, P. Mohammadi, M. Keshavarzi, M.H. Saghi, M. Mohammadi, A. Shams, M. Aghamohammadhasan (2021) Simultaneous determination of organophosphorus pesticides residues in vegetable, fruit juice, and milk samples with magnetic dispersive micro solid-phase extraction and chromatographic method; Recruitment of simplex lattice mixture design for optimization of novel sorbent composites. Analytica Chimica Acta 338802.

[6] P. Sun, S. Zheng, R. Yan, Y. Lian (2022) Determination of organophosphorus pesticides using solid-phase extraction followed by gas chromatography–mass spectrometry. Journal of Chromatographic Science 60 1-6.

[7] X. Zhang, Z. Li, Y. Wang, S. Zhang, X. Zang, C. Wang, Z. Wang (2023) Preparation of black phosphorus nanosheets/zeolitic imidazolate framework nanocomposite for high-performance solid-phase microextraction of organophosphorus pesticides. Journal of Chromatography A 1708 464339.

[8] K. Seebunrueng, S. Tamuang, P. Jarujamrus, S. Saengsuwan, N. Patdhanagul, Y. Areerob, S. Sansuk, S. Srijaranai (2024) Eco-friendly thermosensitive magnetic-molecularly-imprinted polymer adsorbent in dispersive solid-phase microextraction for gas chromatographic determination of organophosphorus pesticides in fruit samples. Food Chemistry 430 137069.
